# Design and Optimization of Novel Competitive, Non-peptidic, SARS-CoV-2 M^pro^ Inhibitors

**DOI:** 10.1021/acsmedchemlett.3c00335

**Published:** 2023-09-28

**Authors:** Leon Jacobs, Aletta van der Westhuyzen, Nicole Pribut, Zackery W. Dentmon, Dan Cui, Michael P. D’Erasmo, Perry W. Bartsch, Ken Liu, Robert M. Cox, Sujay F. Greenlund, Richard K. Plemper, Deborah Mitchell, Joshua Marlow, Meghan K. Andrews, Rebecca E. Krueger, Zachary M. Sticher, Alexander A. Kolykhalov, Michael G. Natchus, Bin Zhou, Stephen C. Pelly, Dennis C. Liotta

**Affiliations:** ⊥ Department of Chemistry, Emory University, Atlanta, Georgia 30322, United States; ∥ COVID-19 Emergency Response, Centers for Disease Control and Prevention, Atlanta, Georgia 30329, United States; # Center for Translational Antiviral Research, Institute for Biomedical Sciences, Georgia State University, Atlanta, Georgia 30303, United States; ∇ Emory Institute for Drug Development, Emory University, Atlanta, Georgia 30322, United States

**Keywords:** SARS-CoV-2, COVID-19, main protease, FEP+

## Abstract

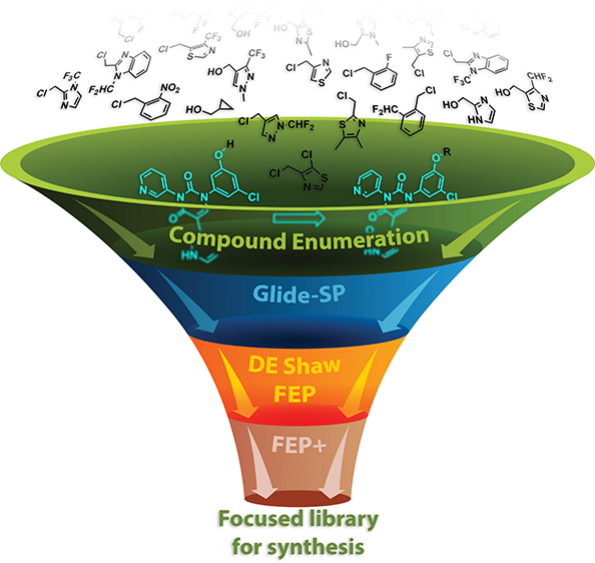

The SARS-CoV-2 main protease (M^pro^) has been proven to be a highly effective target for therapeutic intervention, yet only one drug currently holds FDA approval status for this target. We were inspired by a series of publications emanating from the Jorgensen and Anderson groups describing the design of potent, non-peptidic, competitive SARS-CoV-2 M^pro^ inhibitors, and we saw an opportunity to make several design modifications to improve the overall pharmacokinetic profile of these compounds without losing potency. To this end, we created a focused virtual library using reaction-based enumeration tools in the Schrödinger suite. These compounds were docked into the M^pro^ active site and subsequently prioritized for synthesis based upon relative binding affinity values calculated by FEP+. Fourteen compounds were selected, synthesized, and evaluated both biochemically and in cell culture. Several of the synthesized compounds proved to be potent, competitive M^pro^ inhibitors with improved metabolic stability profiles.

Severe acute respiratory syndrome coronavirus-2 (SARS-CoV-2), the causative agent of the COVID-19 pandemic,^1^ remains problematic, despite the current availability of several vaccines. Moreover, the effectiveness of currently available vaccines is waning as newer SARS-CoV-2 variants emerge.^2^ Thus, there is an urgent, ongoing need for effective, orally bioavailable treatment options. Currently, the FDA has approved three direct-acting antiviral treatments, namely Veklury (remdesivir), Lagevrio (molnupiravir), and Paxlovid (nirmatrelvir and ritonavir). Unfortunately, all three treatments have shortcomings. The first of the viral polymerase substrates, remdesivir, is not orally bioavailable and must be administered intravenously in a hospital setting. Consequently, the drug is mostly used for patients already exhibiting severe COVID-19 symptoms and, in this setting, is of limited efficacy.^3^ Molnupiravir, which is also a substrate for the viral polymerase, should not be administered to pregnant woman, as it may cause fetal harm.^4^ Finally, nirmatrelvir, the first approved inhibitor targeting the viral main protease (M^pro^), must be co-administered with ritonavir (a CYP3A4 inhibitor) to improve its pharmacokinetic properties.^5^ The incorporation of a CYP3A4 inhibitor is sub-optimal for patients on other chronic medications that are metabolized by CYP3A4. Mention must also be made of Xocova (ensitrelvir, currently approved in Japan), another SARS-CoV-2 M^pro^ inhibitor, which has been demonstrated to be highly efficacious,^6^ further highlighting the importance of the main protease as a viral target. Unfortunately, drugs targeting viral proteases are often challenged with the development of resistance, and it would seem that this is, indeed, the case for the SARS-CoV-2 M^pro^, as strains exhibiting some resistance to nirmatrelvir and ensitrelvir have recently been reported.^7^ Thus, there remains an ongoing need to develop next-generation SARS-CoV-2 M^pro^ inhibitors capable of circumventing resistance-causing mutations.

In our quest to discover novel SARS-CoV-2 M^pro^ inhibitors, we sought to follow the path less traveled, by designing inhibitors that were neither peptidic nor covalent modifiers. Peptide-like compounds are often highly polar and are also susceptible to metabolic degradation, both attributes often leading to poor oral bioavailability. At the start of our project, the vast majority of inhibitors being published emanated from earlier work carried out from SARS-CoV research in the early 2000s and thus were almost entirely composed of peptidic compounds (case in point, nirmatrelvir). However, we were inspired by a series of four publications originating from the Jorgensen and Anderson groups,^8−11^ as these compounds were far more drug-like, and we also saw potential for their further improvement. In this work, these groups, with their exceptional strengths in molecular modeling techniques and pharmacologic evaluations, initially carried out a virtual screening of around 2000 approved drugs in search of a viable starting point.^8^ Of the results, 17 compounds were chosen for evaluation in a kinetic M^pro^ inhibition assay, and, remarkably, 14 compounds exhibited inhibition, some with IC_50_ values as low as 5 μM. Within this set, perampanel **1** (Figure 1) was selected (even though it was not the most potent compound), as its simple structure was amenable to further optimization by molecular modeling and it held appeal in terms of synthetic tractability. Through a remarkable feat of modeling ingenuity, the compounds rapidly evolved from the poorly potent **1** to alternate pyridone core-containing compounds such as **2**,^9,11^ which demonstrated a 10-fold improvement in potency. Subsequent expansion into the S4 pocket, as well as improved electrostatic interactions obtained within the S1′ pocket obtained with compounds of type **3** possessing a uracil moiety,^10^ resulted in a final set of compounds exhibiting enzymatic IC_50_ values in the low nM range. Unfortunately, however, the uracil group, although highly beneficial for binding efficacy, would prove to be a liability, as its highly polar nature was believed to be the cause of the poor performance of this compound series in whole-cell antiviral assay studies. This hypothesis was validated by methylation at the N1 nitrogen, leading to compounds of the type **4**, reducing the polarity and improving their performance in the whole cell antiviral assay. However, we envisaged that the crucial N1-methyl may well be a metabolic liability, and, curious about the stability of **4**, we synthesized this compound and subjected it to our in-house human liver microsomal stability assay. Indeed, **4** exhibited a half-life (*t*_1/2_) of just 7.2 min.

**Figure 1:**
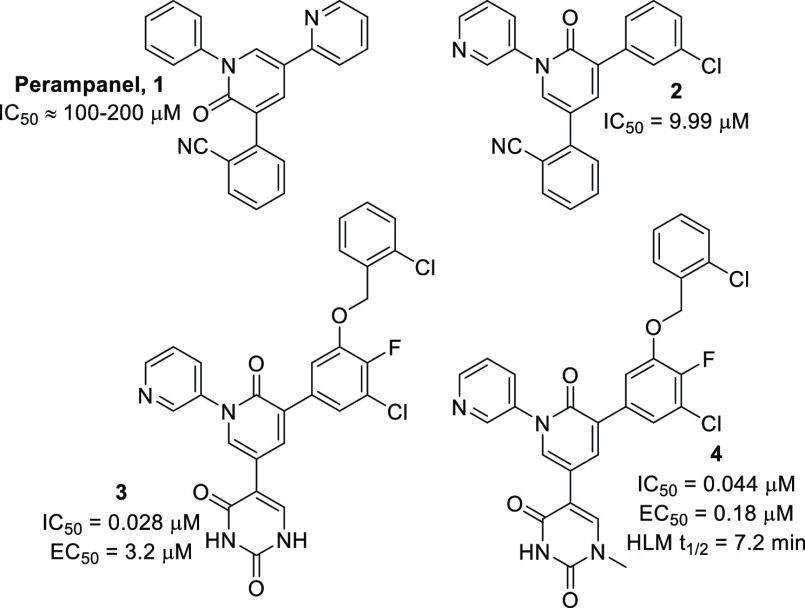
Evolution of the Jorgensen compounds from poorly potent perampanel to highly potent derivatives.

Given our concerns regarding the potential metabolic liability of the methylated uracil moiety (occupying the S1′ pocket), we adopted a slightly different design strategy. We envisioned that it could be replaced by a pyridone, albeit with the loss of one of the three electrostatic interactions that occur with Thr26 in the S1′ pocket and the catalytic thiol of Cys145 (Figure 2A). A docking study of this newly envisaged compound quickly revealed that the lost electrostatic interaction could easily be recovered with the addition of a carbonyl functionality to the existing central pyridone ring, effectively converting the core of the structure to a uracil group (Figure 2B,C). Furthermore, with the uracil moiety now occupying the core of the structure, neither nitrogen possesses an acidic proton, circumventing the problem of ionization and, consequently, poor membrane permeability. Analysis of these structures by free energy perturbation methods (FEP+)^12^ to calculate relative binding free energy values (using the Jorgensen–Anderson structure from PDB 7N44)^10^ revealed that, of the two possible pyridones we could synthesize for the S1′ pocket, the 2-pyridone (Figure 2B) should be substantially better than the 4-pyridone (Figure 2C).

**Figure 2:**
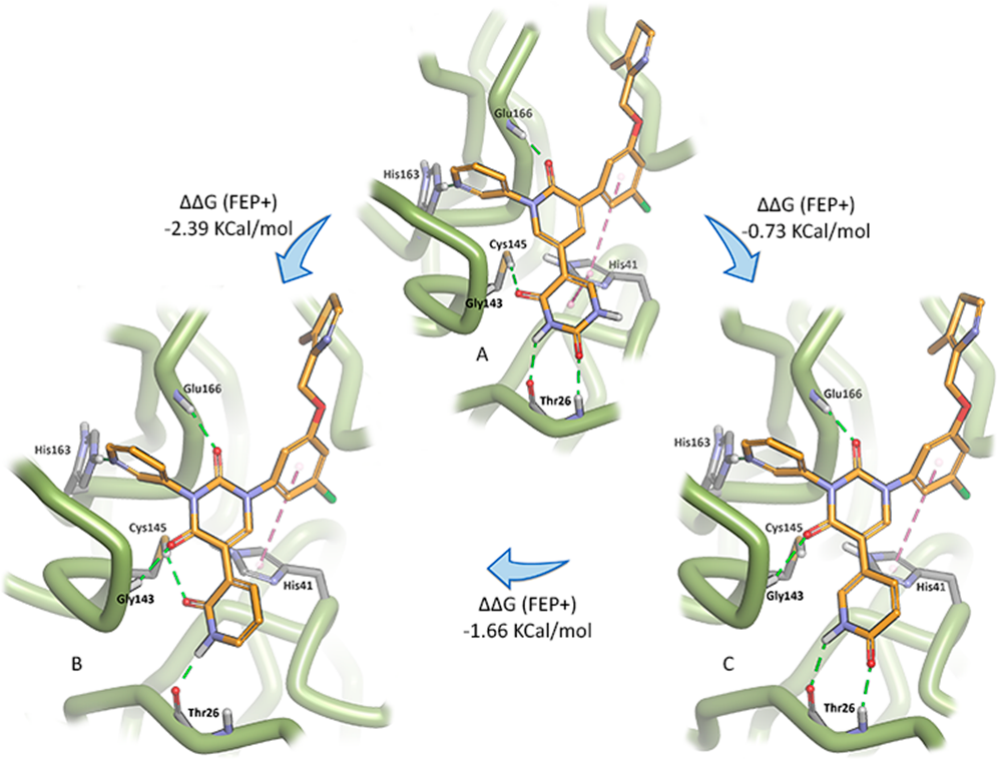
An example of a highly potent Jorgensen compound (A) and conversion of this compound to the 2-pyridone (B), which by relative binding free energy calculations (FEP+) performed significantly better than the alternate 4-pyridone (C).

Having settled on a general structure including the 4-pyridone moiety for the S1′ pocket, the 3-pyridine for the S1 pocket, a uracil as the core motif, and the meta-chlorophenyl for the S2 pocket, we now turned our attention to suitable groups for the S4 pocket (Figure 3). This pocket is essentially hydrophobic, with very few opportunities for electrostatic interactions, yet the judicious incorporation of appropriate small aliphatic, aryl, and heteroaryl groups has the potential to boost the potency of an inhibitor by an order of magnitude.^10^ In order to optimize our synthetic approach to accommodate a late-stage diversification strategy, we planned on arriving at phenol **5**, which would allow us to incorporate a wide range of S4 pocket moieties while minimizing synthetic efforts. With this in mind, we embarked upon a focused virtual screening campaign to identify suitable small fragments which could be purchased (to speed up our program) and were amenable to phenol attachment by S_N_2 substitution of a suitable halide or a Mitsunobu reaction, which broadened our options to now also include suitable alcohol-containing building blocks. In this exercise, over 1500 small, commercially available building blocks were identified, and a library of final compounds was created *in silico* using the Schrödinger reaction-based enumeration tool. These compounds were then docked (Glide-SP) and ranked by docking score as well as by visual inspection, leading to a list of approximately 100 compounds which were then ranked more rigorously by determining relative binding free energy values using free energy perturbation methods (Desmond, D.E. Shaw Research Group).^13^ Finally, relative binding free energy values were determined once again for a final shortlist of 20 compounds using Schrödinger’s FEP+, leading to the identification of 14 compounds for synthesis, which included 2 compounds with the predicated unfavorable 4-pyridone system to validate our modeling hypothesis (Table 1).

**Figure 3:**
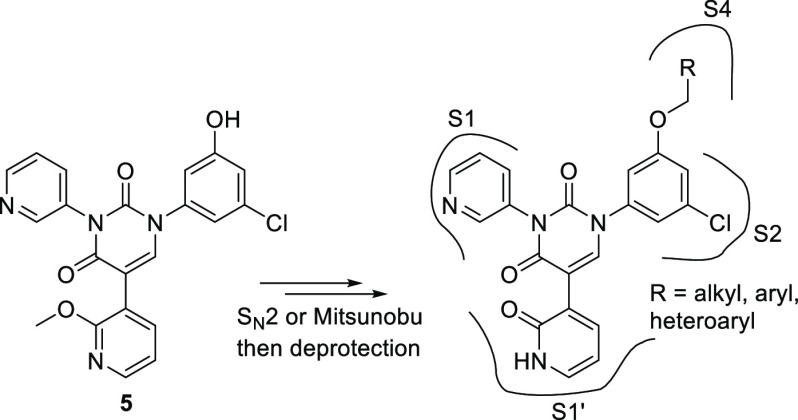
We envisaged being able to attain phenol **5** as our point of late-stage diversification. Various purchasable R-groups amenable to attachment by S_N_2 substitution (chlorides, bromides, iodides) or by way of a Mitsunobu reaction (alcohols) were scrutinized by modeling for optimal S4 pocket occupancy (Glide-SP) and by free energy perturbation methods (FEP+) to calculate relative binding energies for compound ranking.

**Table 1:** Enzyme Inhibition (IC_50_), SARS-CoV-2 Antiviral Activity (EC_50_), and Cellular Toxicity (CC_50_)

Compound	IC_50_^a^ (μM)	EC_50_^b^ (μM)	CC_50_^c^ (μM)
**7**	1.59	>20	>100
**6**	0.28	0.5 (0.4–0.6)	>100
**51**	0.12	>20	>100
**35**	0.10	0.82 (0.7–1)	>100
**36**	0.055	1.35 (1.1–1.6)	>100
**37**	0.043	2.4 (2.1–2.6)	>100
**38**	0.021	1.89 (1.0–3.4)	>100
**39**	0.017	5.0 (4.1–5.9)	>100
**40**	0.016	10.2 (7.6–14)	>100
**41**	0.013	6.76 (4.8–9.2)	>100
**42**	0.012	19.3	>100
**43**	0.0086	0.13 (0.11–0.14)	>100
**44**	0.0056	3.3 (2.9–3.7)	>100
**45**	0.012	0.82 (0.7–0.9)	>100
**3**	0.026 (lit.^10^ 0.028)	–	–
S-217622	–	0.23 (0.21–0.26)	–

a SARS-CoV-2 recombinant protease enzymatic assay.

b Cellular antiviral activity assay against SARS-CoV-2 with recombinant SARS-CoV-2-Nluc reporter virus in VeroE6/TMPRSS2 cells. Parentheses denote 95% confidence intervals for EC_50_ calculations.

c Cellular cytotoxicity.

Before proceeding with the synthesis of compounds expanding into the S4 pocket, we decided to validate our modeling results pertaining to the 2- and 4-pyridone options. To this end, we first opted to test our hypothesis using the simpler derivatives **6** and **7** (Figure 4), which do not possess any S4 pocket functionality. Certainly, relative binding energy analysis by FEP+ suggested once again that 2-pyridone derivative **6** should be more effective than its geometric isomer, **7**.

**Figure 4:**
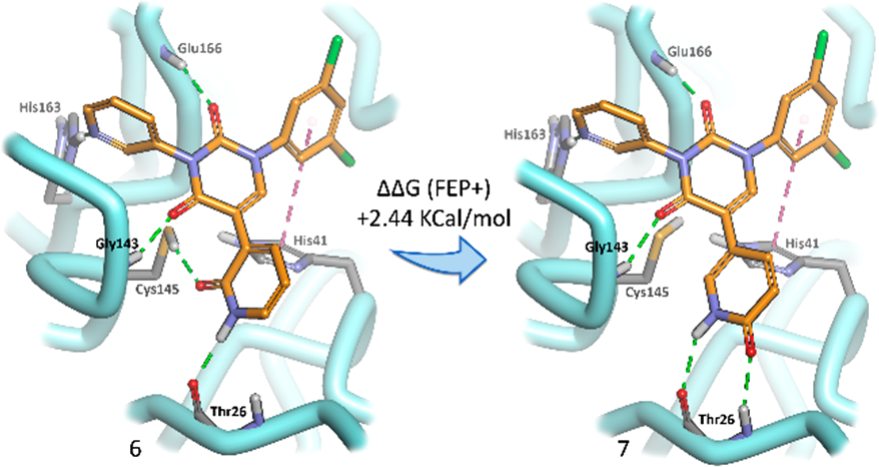
Simpler compounds (**6** and **7**) not containing an S4 binding pocket moiety were evaluated by FEP+ and synthesized to thoroughly investigate which pyridone would be most effective in the S1′ pocket, as this would set the stage for further synthesis.

Synthesis of **6** commenced with a Suzuki cross-coupling reaction between 3-bromo-2-methoxypyridine **8** and the boronic acid **9**, affording **10** in excellent yield (Scheme 1). At this point, the uracil core was revealed by debenzylation under hydrogenative conditions, affording **11** in quantitative yield. It was at this stage that we ran into synthetic difficulties. We initially envisaged that we would be able to carry out a Chan–Lam coupling between 3-pyridyl boronic acid **14** and preferably the desired N3 of our uracil derivative, **11**.^14^ Unfortunately, however, the required selectivity was not attained under these conditions, and in fact, we obtained a slight preponderance of the undesired reaction at N1 (as determined by X-ray crystallography). Fortunately, a survey of the literature revealed just one single article which rescued our planned synthetic route. Facing a similar chemoselectivity problem, Barnes et al. developed methodology based on the copper-catalyzed Ulmann–Goldberg reaction, employing *N*-(2-cyanophenyl)picolinamide as the ligand in a copper-mediated cross-coupling reaction.^15^ Under these conditions, they achieved a high selectivity for coupling at N1. With this promising new strategy in mind, we switched the order of our planned coupling reactions and now opted to install the dichlorophenyl moiety first, at N1. Indeed, under the conditions developed by Barnes et al., we achieved coupling exclusively at N1 (determined by X-ray crystallography) when reacting **12** with **11**, albeit in modest yield, thereby arriving at **13**. With only N3 now available for the reaction, we were able to successfully install the pyridyl moiety using **14**, forming **15**. Finally, the pyridone for the S1′ pocket was revealed after demethylation using TMSCl and NaI, affording the target compound **6**.

**Scheme 1:**
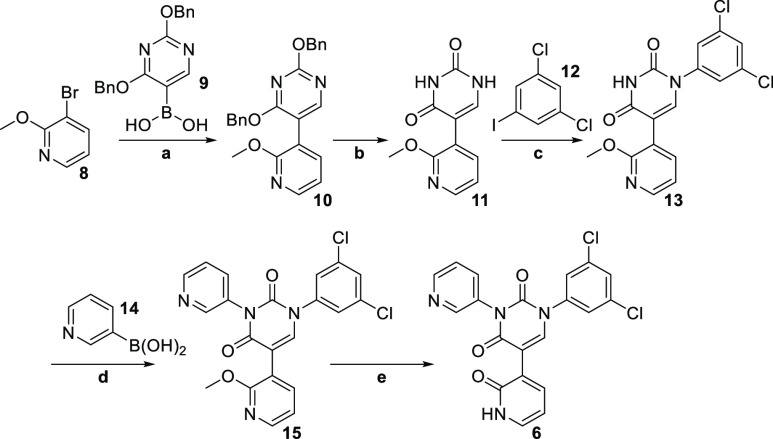
**Reagents and conditions:** a) Pd(PPh_3_)_4_, NaHCO_3_, **9**, DME/water, 93%; b) 10% Pd/C, H_2_, MeOH/THF (1:1), 92%; c) CuI, K_3_PO_4_, *N*-(2-cyanophenyl)picolinamide, **11**, DMSO, 41%; d) Cu(OAc)_2_, TMEDA, **14**, DMSO, 95%; e) TMSCl, NaI, MeCN, 62%.

The alternate pyridone derivative **7** was similarly synthesized by starting with 5-bromo-2-methoxypyridine **16** (Scheme 2).

**Scheme 2:**
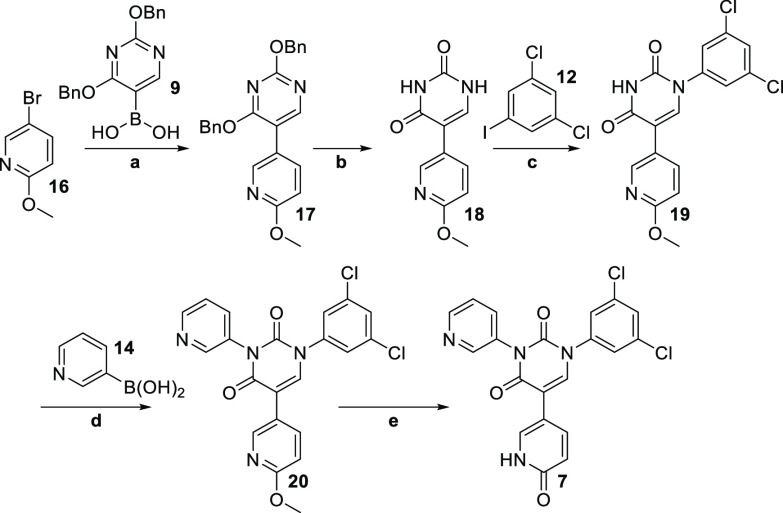
**Reagents and conditions:** a) Pd(PPh_3_)_4_, NaHCO_3_, **9**, DME/water, 86%; b) 10% Pd/C, H_2_, MeOH/THF (1:1), 27%; c) CuI, K_3_PO_4_, *N*-(2-cyanophenyl)picolinamide, **11**, DMSO, 35%; d) Cu(OAc)_2_, TMEDA, **14**, DMSO, 81%; e) TMSCl, NaI, MeCN, 94%.

Compounds **6** and **7** were then evaluated in a SARS-CoV-2 recombinant protease enzymatic assay, and gratifyingly, the IC_50_ values (0.276 μM and 1.585 μM, respectively, Table 1) corroborated our FEP+ relative binding energy evaluation studies, with the 2-pyridone derivative, **6**, being considerably more potent than the 4-pyridone derivative, **7**.

Having confirmed the preferred pyridone for the S1′ pocket, we next set about synthesizing the series containing groups extending into the S4 pocket. Thus, starting from **11** (Scheme 3), installation of the benzyl-protected phenol moiety for the S2 pocket using **21** (itself readily synthesized from 3-chloro-5-iodophenol) was carried out regioselectively according to the procedure developed by Barnes et al.^15^ as described earlier, providing exclusively **22**, though often in modest yields. At this point, another copper-mediated coupling of 3-pyridyl boronic acid under Chan–Lam conditions provided **23** in good yields. Debenzylation of **23** under standard hydrogenation conditions afforded the key phenol **5**, ready for attachment of our assortment of S4 pocket moieties, as prioritized by the FEP+ modeling described earlier. Thus, the corresponding halides or alcohols were reacted with phenol **5** in the presence of potassium carbonate or under Mitsunobu conditions, respectively, leading to desired penultimate compounds **24**–**34** in moderate to excellent yields. It should be mentioned that all of these derivatives were commercially available, with the exception of the R_3_, R_10_, and R_11_ derivatives (Scheme 3), which we needed to construct (see Supporting Information). Finally, the crucial S1′ pocket pyridones were revealed by treating each compound with TMSCl and NaI, affording target compounds **35**–**45** in generally moderate yields.

**Scheme 3:**
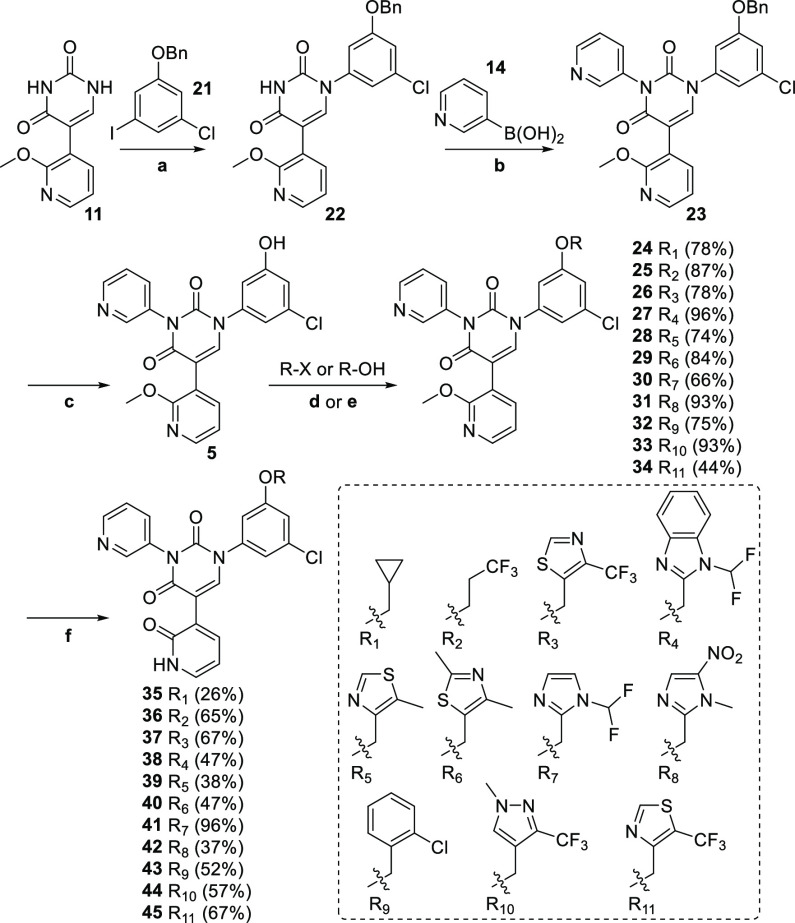
**Reagents and conditions:** a) CuI, K_3_PO_4_, *N*-(2-cyanophenyl)picolinamide, **21**, DMSO, 46%; b) Cu(OAc)_2_, TMEDA, **14**, DMSO, 90%; c) 10% Pd/C, H_2_, MeOH/THF (1:1), 95%; d) K_2_CO_3_, R-X, DMF; e) DIAD, PPh_3_, R-OH, THF; f) TMSCl, NaI, MeCN.

In parallel with this effort, we also synthesized the alternate 4-pyridone version of **39**, namely **51** (Scheme 4), in order to verify that, even with an attached S4 moiety, the FEP+ analysis was still correct in predicting that the 2-pyridone derivatives would generally be more potent than the 4-pyridone derivatives. The synthetic route followed the identical path as for the 2-pyridone derivatives, even though the benzyl protection and deprotection steps were not technically necessary (since our intention was to make a single compound). Indeed, it would have been possible to directly couple **49** to 3-chloro-5-iodophenol and simply use that at the start of the synthesis. However, we intentionally followed the same route as previously described, thereby arriving at the phenol **48**, so that we would have it available, should it prove necessary to synthesize more derivatives in this series. Thus, target compound **51** (being the alternate pyridone derivative to **39**) was synthesized relatively uneventfully, starting from **18**.

**Scheme 4:**
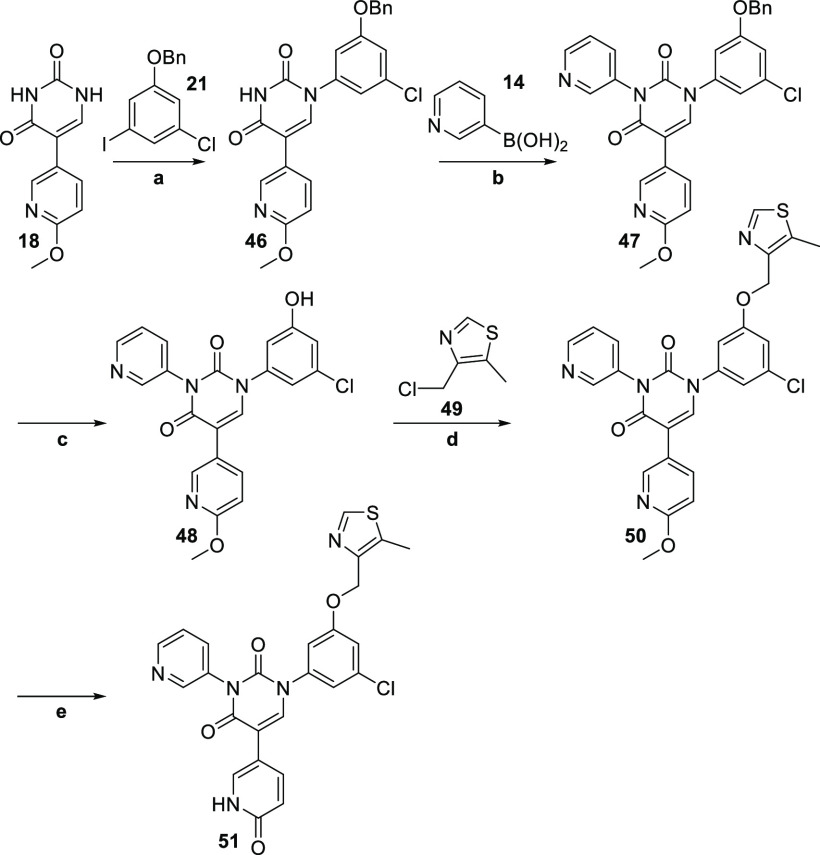
**Reagents and conditions:** a) CuI, K_3_PO_4_, *N*-(2-cyanophenyl)picolinamide, **21**, DMSO, 18%; b) Cu(OAc)_2_, TMEDA, **14**, DMSO, 87%; c) 10% Pd/C, H_2_, MeOH/THF (1:1), 85%; d) K_2_CO_3_, **49**, DMF, 84%; e) TMSCl, NaI, MeCN, 45%.

With compounds **35**–**45** and **51** in hand, we were able to assess their efficacy in a SARS-CoV-2 recombinant protease enzymatic assay (Table 1, IC_50_). From the respective IC_50_ values, the *K*_i_ values could be determined, allowing us to compare the calculated Gibbs free energy of binding (FEP+) with the experimentally derived values. Pleasingly, an excellent correlation was found between the calculated and experimental results (Figure 5), emphasizing the utility of modern computational drug design methods. As predicted by FEP+, the 4-pyridone derivative **51** did, indeed, turn out to be a poorer inhibitor than its geometric isomer, **39**. Furthermore, two of the compounds, **43** and **44**, had IC_50_ values in the single-digit nanomolar range. We also synthesized and tested one of the most potent compounds from the Jorgensen group, **3**,^10^ and were pleased to discover that our design yielded similarly potent compounds and that our enzymatic assay results were in good agreement with those obtained by the Jorgensen and Anderson groups.

**Figure 5:**
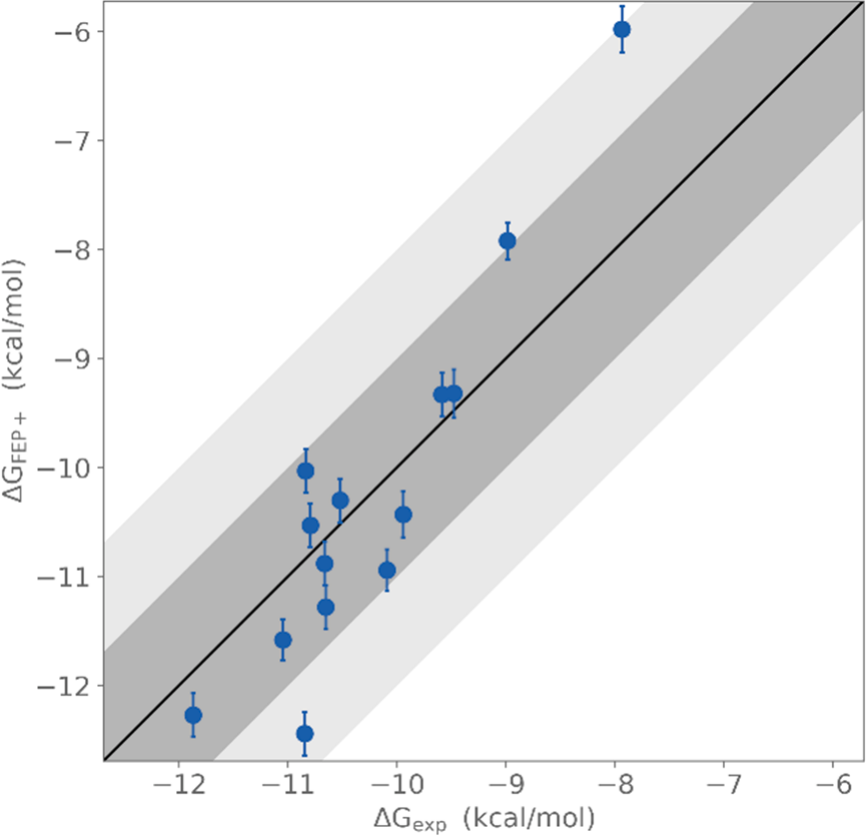
Calculated binding free energy values (Δ*G*_FEP+_) plotted against experimentally determined binding free energy values (Δ*G*_EXP_).

The compounds were then evaluated in a cellular antiviral assay (EC_50_), revealing compounds **43**, **45**, and **6** (somewhat surprisingly since this compound does not contain an S4 pocket moiety) as potent compounds exhibiting sub-micromolar EC_50_ values, comparable to that of the promising new Shionogi SARS-CoV-2 M^pro^ inhibitor, S-217622 (ensitrelvir).^16^

Having established that our design modifications afforded compounds of comparable potency to those emanating from the Jorgensen and Anderson groups, it was now time to address the key question and, indeed, the motivation behind the entire study. Namely, did our design modification afford compounds with an improved metabolic profile? To this end, we identified several key compounds and assessed their stability in human, mouse, and rat liver microsomes (Table 2). For comparison purposes, the potent Jorgensen–Anderson compound **4** (Figure 1) was also included in this study. All four compounds tested in our series exhibited superior liver microsomal stability profiles compared to **4**. However, intriguingly, all our compounds containing an aromatic S4 pocket moiety (**41**, **43**, and **45**) were significantly less metabolically stable than our compound **36**, which contains an aliphatic S4 pocket moiety. This observation prompted us to re-evaluate our hypothesis regarding the observed liver microsomal instability of Jorgensen–Anderson compound **4**. Indeed, metabolite identification studies (not available at the start of our project) revealed that loss of the uracil N1 methyl was, in fact, not the major metabolite, but rather it was loss of the ortho-chlorobenzyl moiety (SI, Figure S16). This same phenomenon was observed when we similarly identified the microsomal metabolites for compound **43**. Furthermore, the inferior stability of the Jorgensen–Anderson compound **4** compared to **43** (even though they both contain the same S4 pocket moiety) is attributed to the fact that, although the loss of the benzylic group is the major metabolite for **4**, some loss of the methyl at N1 is also observed. Unfortunately, in our series, compounds with an aromatic S4 pocket moiety were generally found to be more potent than their aliphatic counterparts (Table 1), leading to somewhat of a conundrum in selecting a compound with which to proceed forward that exhibited a good balance of potency and stability.

**Table 2:** Liver Microsomal Stability Studies for Selected Compounds, Showing Percentage Remaining after 30 min and *t*_1/2_

	**4**	**36**	**41**	**43**	**45**
HLM (*t*_1/2_)	4.9% (5.6)	91.7% (>30)	70.7% (>30)	48.0% (27.8)	19.5% (13)
MLM (*t*_1/2_)	0.7% (2.4)	73.3% (>30)	22.8% (14.7)	4.0% (3.0)	14.4% (11)
RLM (*t*_1/2_)	1.4% (4.8)	86.4% (>30)	90.2% (>30)	26.6% (19.8)	30.9% (18)

Having established that **36** exhibited reasonable potency in our enzymatic and whole-cells assay, and that it was our most stable compound in the liver microsomal studies, we decided to push forward with this compound to determine its oral bioavailability in rats. To this end, **36** was studied in male Sprague–Dawley rats following a single intravenous dose at 0.25 mg/kg and an oral dose at 0.5 mg/kg (SI, Table S18). The results of this study indicated a peak plasma concentration at 1.67 h, suggesting rapid absorption. Unfortunately, a modest oral bioavailability (41%), despite low plasma clearance (2.49 mL/min/kg) and rapid uptake, was indicative of poor compound solubility. This problem was further highlighted in a dose escalation study (10 mg/kg and 100 mg/kg PO), where it was observed that the 10-fold increase in dose resulted in a only 2-fold increase in AUC (SI, Table S19).

In summary, inspired by the potent SARS-CoV-2 M^pro^ inhibitors developed by the Jorgensen and Anderson groups, we set out to improve upon their design by changing the uracil moiety occupying the S1′ pocket to a pyridone. Analysis of the new design by FEP+ suggested that our compounds would be as effective, and indeed, this turned out to be the case. Furthermore, FEP+ proved to be an extremely valuable tool in prioritizing compounds for synthesis, and an excellent correlation was obtained between the predicted binding free energy values and those later calculated from measured IC_50_ results. Although these new compounds did prove to be metabolically more stable than the highly potent Jorgensen–Anderson compound **4**, they still present some challenges. In particular, aqueous solubility remains a problem, despite the polar nature of these compounds. Nevertheless, the design and development of novel, potent SARS-CoV-2 Mpro inhibitors remain an important priority. The shortcomings of the only FDA-approved M^pro^ inhibitor (nirmatrelvir) have been mentioned above, and the promising new M^pro^ inhibitor being developed by Shionogi (ensitrelvir) is a strong CYP3A inhibitor that may present serious adverse drug–drug interactions for patients on other chronic medication.^17^ Furthermore, another newly emerging opportunity in this area lies in the development of SARS-CoV-2 M^pro^ inhibitors capable of overcoming issues pertaining to resistant viral variants, which are just coming to the fore for both ensitrelvir and nirmatrelvir.^7,18^

## Supporting Information Available

Experimental procedures, assay details, pharmacokinetic experiments, and modeling methodology (PDF)

## Abbreviations

SARS-CoV-2: severe acute respiratory coronavirus-2
M^pro^: main protease
FEP: free energy perturbation
TMSCl: trimethylsilyl chloride
DIAD: diisopropyl azodicarboxylate
TEMPO: 2,2,6,6-tetramethylpiperidine-1-oxyl
IC_50_: half-maximal inhibitory concentration
EC_50_: half-maximal effective concentration
DMSO: dimethyl sulfoxide
DMF: dimethylformamide
DME: dimethoxyethane.
TMEDA: tetramethylethylenediamine
CC_50_: concentration of test compound required to reduce cell viability by 50%
