# Supplementary material for: Design and Optimization of Novel Competitive, Non-peptidic, SARS-CoV-2 Mpro Inhibitors
Source: ACS Med Chem Lett. 2023 Sep 28;14(10):1434–40. doi: 10.1021/acsmedchemlett.3c00335 (PMC10577883; doi:10.1021/acsmedchemlett.3c00335)

Supporting Information

Design and optimization of novel competitive, non-peptidic, SARS-CoV-2 Mpro inhibitors

Leon Jacobs[†ϒ], Aletta van der Westhuyzen[†ϒ], Nicole Pribut[†], Zackery W. Dentmon[†a], Dan Cui[§], Michael P. D'Erasmo[†b], Perry W. Bartsch[†], Ken Liu[†], Robert M. Cox[¥]; Sujay Greenlund[¥], Richard K. Plemper[¥], Deborah Mitchell[‡], Joshua Marlow[‡], Meghan K. Andrews[‡], Rebecca E. Krueger[‡], Zachary M. Sticher[‡], Alexander A. Kolykhalov[‡], Michael G. Natchus[‡], Bin Zhou[§], Stephen C. Pelly[†]* and Dennis C. Liotta[†]*.

[†] Department of Chemistry, Emory University College of Arts and Sciences, Atlanta, GA, USA.

[§] COVID-19 Emergency Response, Centers for Disease Control and Prevention, Atlanta, GA, USA.

[¥] Center for Translational Antiviral Research, Institute for Biomedical Sciences, Georgia State University, Atlanta, GA, USA.

[‡] Emory Institute for Drug Development, Emory University Atlanta, GA, USA.

Table of Contents

Synthesis General Procedures and Schemes S2

General S2

General Procedures S3

Scheme S1: Synthesis towards target compound **6** S4

Scheme S2: Synthesis towards target compound **7** S6

Scheme S3: Synthesis towards target compounds **35–45**..S9

Scheme S4: Synthesis towards target compound **51** S17

Scheme S5: Synthesis towards precursors **55** and **57** S19

Molecular Modelling S20

Enzymatic and Whole Cell Assay Procedures S21

Enzymatic (IC_{50}) Assay Protocol S21

Whole Cell (EC_{50}) Assay Protocol S21

Metabolic Stability Assay S22

Liver Microsome Stability Assay Protocol S22

Instrumentation and LC-MS/MS Method development S23

Table S1: Scan parameters and corresponding transitions in MRM mode S23

Liver Microsome Stability Data of Selected Compounds S24

Table S2: Liver microsome stability data as % remaining and half-life S24

Figures S1-S15 and Tables S3-S17 ....S24

Metabolite Identification Studies S34

Pharmacokinetic Studies S36

Table S18: PK data of **36** after a single IV and PO administration...........................S36

Figure S17: Plasma concentration-time profiles of **36** following a single IV (0.25 mg/kg) and PO (0.5 mg/kg) dose...S36

Table S19: PK parameters of **36** in rat plasma after a single oral dose of **36** at 10 mg/kg or 100 mg/kg S37

Figure S18: Average concentrations of **36** in rat plasma after a single oral dose of **36** at 10 mg/kg or 100 mg/kg S37

Figure S19: Figure of all final compounds tested........... ....S38

Synthesis General Procedures and Schemes:

General

Unless otherwise indicated, all reactions requiring anhydrous conditions were conducted in oven dried or flame-dried glassware using distilled and degassed solvents under a positive pressure of dry argon. General chemicals and reagents were obtained from commercial suppliers and were used without further purification. Typically, reaction progress was monitored by either thin layer chromatography (TLC) using silica-precoated glass plates

(Merck KGaA; silica gel 60 F_{254}, 0.25 mm thickness) or liquid chromatography–mass spectrometry (LC-MS) on an Agilent Technologies 6100 quadrupole instrument equipped with UV detection at 254 and 210 nm and Agilent C18 XDB eclipse column (50 mm x 4.6 mm, 3.5 µM). Automated flash column chromatography was performed using Teledyne ISCO CombiFlash Companion systems with silica gel-packed columns or RediSepRf reverse-phase C18 gold columns (Teledyne Isco). NMR spectra (^{1}H, ^{13}C, and ^{19}F) were obtained using either a Varian INOVA 600 MHz spectrometer, a Varian INOVA 500 MHz spectrometer, a Varian INOVA 400 MHz spectrometer, a Varian VNMR 400 MHz spectrometer, a Bruker 400 MHz spectrometer, or a Bruker 600 MHz spectrometer. NMR samples were prepared and processed in deuterated chloroform ($CDCl_3$, residual solvent peaks: ^{1}H = 7.26 ppm, ^{13}C = 77.16 ppm) or deuterated methanol (CD_3OD, residual solvent peaks: ^{1}H = 3.31 ppm, ^{13}C = 49.0 ppm) or deuterated DMSO (DMSO, residual solvent peaks: ^{1}H = 2.50 ppm, ^{13}C = 39.52 ppm). The residual chloroform (or TMS if present) or methanol or H_2O peak in ^{1}H NMR was used as an absolute reference for ^{19}F NMR. NMR data were reported to include chemical shifts (δ) reported in ppm, multiplicities indicated as s (singlet), d (doublet), t (triplet), q (quartet), p (pentet), hept (heptet), td (triplet of doublets), m (multiplet), br (broad), coupling constants (*J*) reported in Hz, and integration normalized to 1 atom (H, C, or P). High resolution mass spectrometry (HRMS) was performed by the Emory University Mass Spectrometry Center, directed by Dr. Fred Strobel. Liquid chromatography-mass spectrometry (LC-MS) was performed on an Agilent 1200 HPLC equipped with a 6120 Quadrupole mass spectrometer (ESI-API) eluting with mixtures of HPLC grade MeOH and H_2O (0.1% formic acid) or MeCN and H_2O (0.1% formic acid) using an analytical, reverse-phase, Agilent C18 XDB eclipse column (50 mm x 4.6 mm, 3.5 µM). LC-MS samples were prepared in aqueous solutions of MeOH, or pure MeOH. Final compound purity was assessed using NMR and LC-MS, and the purity of all final compounds reported herein were determined to be ≥95% pure.

General Procedures

General Procedure A:

Into a 100 mL two neck round bottom flask containing argon was placed the respective phenol (1 eq) followed by DMF (15 mL per 500 mg), thus forming a clear to pale yellow solution. To this was added potassium carbonate (3 eq) and then the respective electrophile (R-CH_2-X, where X = Cl, Br or I) (1.3 eq). The reaction was heated to 60 °C for 18 hours, after which time analysis by LCMS typically indicated complete conversion of the starting material. The reaction mixture was diluted with EtOAc (100 mL) and water (100 mL) and the phases were mixed and separated. The aqueous phase was extracted twice with EtOAc (2 x 100 mL) and then the combined organic fractions were washed with brine (100 mL), separated and dried over anhydrous magnesium sulfate. After concentrating *in vacuo* the crude material was purified by column chromatography (EtOAc/Hex) to afford the desired products as white to off-white solids.

General Procedure B:

Into a 2 neck 100 mL round bottom flask filled with argon was placed respective phenol (1 eq) followed by CH_3CN (50 mL for 500 mg), the respective alcohol (1.4 eq) and finally triphenylphosphine (1.4 eq), typically forming a heterogenous mixture. This was cooled to 0 °C and then diisopropyl azodicarboxylate (1.4 eq) was added, often resulting in a homogeneous solution. The reaction was then heated to 55 °C and left to proceed under argon for 18 h. The reaction mixture was diluted with EtOAc (150 mL) and water (150 mL) and the phases were mixed and separated. The aqueous phase was extracted twice with EtOAc (2 x 150 mL) and then the combined organic fractions were washed with brine (150 mL), separated and dried over anhydrous magnesium sulfate. After

concentrating *in vacuo* the crude material was purified by column chromatography (EtOAc/Hex) to afford the desired products as white to off-white solids.

General Procedure C:

Into a 100 mL 2 neck round bottom flask containing argon was placed the methoxy pyridine derivative (1 eq) followed by dry MeCN (30 mL for 500 mg), thus forming a clear solution. To this was added sodium iodide (3 eq) followed immediately by chlorotrimethylsilane (3 eq), resulting in a pale yellow solution. The reaction mixture was heated to 60 °C for 30 min after which time analysis by TLC revealed complete consumption of the starting material. After cooling, the reaction mixture was diluted with EtOAc (150 mL) and 5% sodium thiosulfate solution (150 mL). After thoroughly mixing, the phases were separated, and the aqueous phase was extracted with EtOAc (2 x 100 mL). The combined organic fractions were then washed with brine, dried over anhydrous magnesium sulfate, filtered, and then concentrated *in vacuo*. The crude material was purified by column chromatography (initially EtOAc/Hex, then 3% MeOH/EtOAc) to afford the desired pyridone products as a white to off-white solids.

Scheme S1: Synthesis towards target compound 6

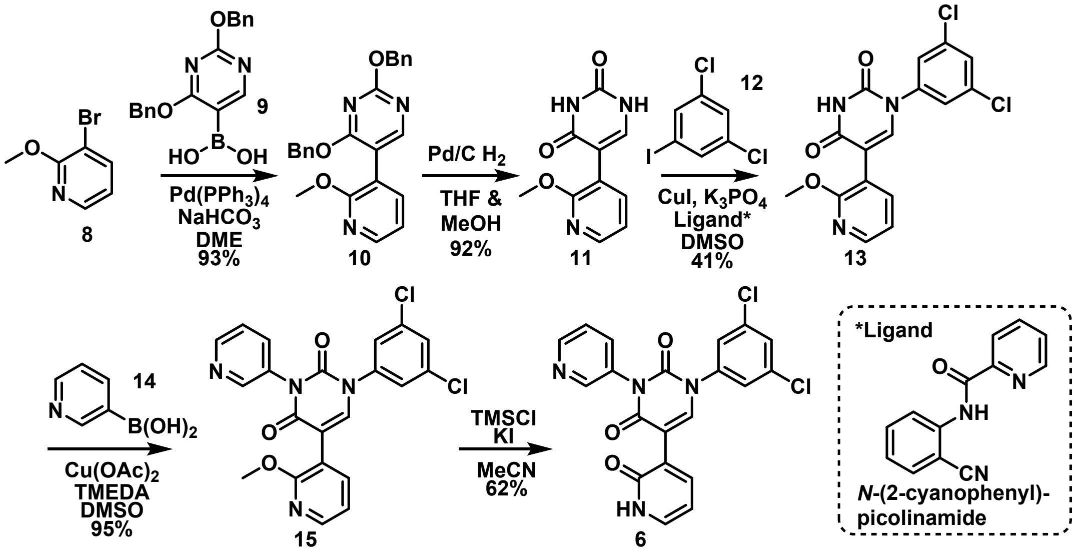

<u>Synthesis of 2,4-Bis(benzyloxy)-5-(2-methoxypyridin-3-yl)pyrimidine (**10**)</u>

Into a 500 mL 3 neck round bottom flask fitted with a condenser, and filled with argon, was placed 3-bromo-2-methoxy-pyridine **8** (2.8 mL, 23 mmol) followed by DME (200 mL), thus forming a clear solution. To this was added (2,4-dibenzyloxypyrimidin-5-yl)boronic acid **9** (9.65 g, 28.7 mmol) which quickly dissolved and then saturated sodium bicarbonate solution was added (150 mL), resulting in an immediate white precipitate forming. The solution was then degassed for 10 minutes by bubbling argon into the mixture and then tetrakis(triphenylphosphine)palladium(0) (5.53 g, 4.79 mmol) was added in one portion. The reaction mixture was heated to reflux under argon resulting in most of the white precipitate solubilising, and thus forming a yellow solution. The reaction was left to proceed for 6 h at reflux and the colour changed to a darker orange colour. After this time, it was cooled and poured into a separating funnel and diluted with EtOAc (400 mL) and water (300 mL). After vigorously shaking, the layers were allowed to separate, and the organic layer was collected. The aqueous

layer was extracted twice more with EtOAc (2 x 200 mL). The organic fractions were combined and dried over anhydrous magnesium sulfate and then filtered. After concentrating to *ca* 300 mL, this organic phase was pulled through a filter bed of silica gel. The silica bed was washed with ethyl acetate. The organic solution was then concentrated *in vacuo* and the crude material purified by column chromatography (EtOAc/Hex) to afford 2,4-dibenzyloxy-5-(2-methoxy-3-pyridyl)pyrimidine **10** (8.86 g, 22.2 mmol, 93% yield) as a white solid.

^{1}H NMR (400 MHz, $CDCl_3$) δ 8.28 (s, 1H), 8.18 (dd, *J* = 5.1, 1.9 Hz, 1H), 7.56 (dd, *J* = 7.3, 1.9 Hz, 1H), 7.52 – 7.48 (m, 2H), 7.42 – 7.27 (m, 8H), 6.94 (dd, *J* = 7.3, 5.0 Hz, 1H), 5.47 (s, 2H), 5.45 (s, 2H), 3.87 (s, 3H).

Synthesis of 5-(2-Methoxypyridin-3-yl)pyrimidine-2,4(1*H*,3*H*)-dione (**11**)

Into a 2 neck 500 mL round bottom flask containing methanol (150 mL) and THF (150 mL) was placed 2,4-dibenzyloxy-5-(2-methoxy-3-pyridyl)pyrimidine **10** (8.10 g, 20.3 mmol) thus forming a clear solution, which was blanketed with argon. To this was then added 10% palladium on carbon (2.16 g, 20.3 mmol) in one portion. A balloon containing hydrogen was fitted to the flask and the flask was purged somewhat with hydrogen. The reaction was heated to 45 °C and was left to proceed for 10 h after which time analysis by LCMS indicated that all of the starting material had been converted to the desired product. The reaction mixture was filtered through celite and concentrated *in vacuo* to afford 5-(2-methoxy-3-pyridyl)-1*H*-pyrimidine-2,4-dione **11** (4.10 g, 18.7 mmol, 92% yield) as a white solid. (Note that earlier attempts to purify this material by column chromatography proved very problematic due to precipitation of the material within the column).

^{1}H NMR (400 MHz, DMSO) δ 11.24 (s, 1H), 11.07 (s, 1H), 8.11 (dd, *J* = 5.0, 1.9 Hz, 1H), 7.69 (dd, *J* = 7.3, 1.9 Hz, 1H), 7.57 (s, 1H), 7.00 (dd, *J* = 7.3, 5.0 Hz, 1H), 3.83 (s, 3H).

Synthesis of 1-(3,5-Dichlorophenyl)-5-(2-methoxypyridin-3-yl)pyrimidine-2,4(1*H*,3*H*)-dione (**13**)

A Schlenk tube was charged with 5-(2-methoxypyridin-3-yl)pyrimidine-2,4(1*H*,3*H*)-dione **11** (483 mg, 2.20 mmol), followed by 1,3-dichloro-5-iodobenzene **12** (500 mg, 1.83 mmol), K_3PO_4 (817 mg, 3.85 mmol), CuI (35 mg, 0.18 mmol) and *N*-(2-cyanophenyl)picolinamide (50 mg, 0.22 mmol). The vessel was evacuated and back filled with argon. DMSO (13 mL) was added and the reaction mixture was degassed for 15 min by bubbling argon into the solution. The reaction was stirred overnight at 60 °C. Once cooled, the reaction solution was diluted with EtOAc and washed with water. The aqueous layer was then extracted with EtOAc (x3). The combined organic layers were then washed with $Cu(OAc)_2$.H2O (x2), 15% NH_4Cl (x2), brine and dried over $MgSO_4$. After filtration and removal of solvent *in vacuo*, the crude material was purified using column chromatography (1-5% MeOH/DCM) to afford 1-(3,5-dichlorophenyl)-5-(2-methoxypyridin-3-yl)pyrimidine-2,4(1*H*,3*H*)-dione **13** (268 mg, 0.736 mmol, 40%) as a white solid.

Rf = 0.48 (5% MeOH/DCM). **^{1}H NMR (400 MHz, DMSO) δ** 10.82 (br s, 1H), 7.21 (dd, *J* = 5.0, 1.9 Hz, 1H), 6.99 (s, 1H), 6.84 – 6.73 (m, 4H), 6.09 (dd, *J* = 7.3, 5.0 Hz, 1H), 2.89 (s, 3H). **^{13}C NMR (101 MHz, DMSO) δ** 162.1, 161.2, 149.8, 146.3, 143.4, 140.6, 140.3, 134.1 (2C), 128.1, 126.4 (2C), 116.8, 116.0, 110.6, 53.4.

Synthesis of 1-(3,5-Dichlorophenyl)-5-(2-methoxypyridin-3-yl)-3-(pyridin-3-yl)pyrimidine-2,4(1*H*,3*H*)-dione (**15**)

To a round-bottomed flask was added 1-(3,5-dichlorophenyl)-5-(2-methoxypyridin-3-yl)pyrimidine-2,4(1*H*,3*H*)-dione **13** (250 mg, 0.687 mmol), followed by 3-pyridinyl boronic acid **14** (253 mg, 2.06 mmol) and DMSO (3 mL). Copper(II) acetate (125 mg, 0.687 mmol) was then added to the reaction solution followed by TMEDA (0.23 mL, 1.5 mmol). The reaction mixture was stirred at 60 °C open to the atmosphere and monitored using LCMS. Once

completed, EtOAc was added and the solution was washed with H_2O (x2), brine and dried over $MgSO_4$. Filtration was then followed by *in vacuo* removal of solvent and purification by column chromatography (EtOAc/Hex) to afford 1-(3,5-dichlorophenyl)-5-(2-methoxypyridin-3-yl)-3-(pyridin-3-yl)pyrimidine-2,4(1*H*,3*H*)-dione **15** as a white solid (286 mg, 0.648 mmol, 95%).

Rf = 0.38 (5% MeOH/DCM). **^{1}H NMR (600 MHz, DMSO) δ** 8.62 (dd, *J* = 4.8, 1.5 Hz, 1H), 8.58 (d, *J* = 2.4 Hz, 1H), 8.20 – 8.16 (m, 2H), 7.85 (ddd, *J* = 8.1, 2.4, 1.6 Hz, 1H), 7.81 – 7.73 (m, 3H), 7.57 (ddd, *J* = 8.1, 4.8, 0.8 Hz, 1H), 7.22 – 7.12 (m, 1H), 7.06 (dd, *J* = 7.3, 5.0 Hz, 1H), 3.85 (s, 3H). **HRMS**: (APCI+) $[M+H]^+$ calc. for $C_{21}H_{15}Cl_2O_3N_4$, 441.0521, observed 441.05146.

Synthesis of 1-(3,5-Dichlorophenyl)-5-(2-oxo-1,2-dihydropyridin-3-yl)-3-(pyridin-3-yl)pyrimidine-2,4(1*H*,3*H*)-dione (**6**)

Reaction carried out according to G*eneral procedure-C*: Using 1-(3,5-dichlorophenyl)-5-(2-methoxypyridin-3-yl)-3-(pyridin-3-yl)pyrimidine-2,4(1*H*,3*H*)-dione **15** (270 mg, 0.614 mmol), NaI (230 mg, 1.53 mmol), TMSCl (166 mg, 1.53 mmol), acetonitrile (24 mL). Afforded 1-(3,5-dichlorophenyl)-5-(2-oxo-1,2-dihydropyridin-3-yl)-3-(pyridin-3-yl)pyrimidine-2,4(1*H*,3*H*)-dione **6** as an off-white solid (162 mg, 0.379 mmol, 62%).

Rf = 0.60 (15% MeOH/DCM); **^{1}H NMR (400 MHz, DMSO) δ** 11.92 (s, 1H), 8.69 (s, 1H), 8.62 (dd, *J* = 4.8, 1.6 Hz, 1H), 8.56 (d, *J* = 2.4 Hz, 1H), 7.99 (dd, *J* = 7.1, 2.1 Hz, 1H), 7.84 (ddd, *J* = 8.1, 2.5, 1.5 Hz, 1H), 7.79 – 7.77 (m (app.t), 1H), 7.75 (d, *J* = 1.9 Hz, 2H), 7.57 (ddd, *J* = 8.1, 4.8, 0.8 Hz, 1H), 7.38 (dd, *J* = 6.4, 2.1 Hz, 1H), 6.30 (dd, *J* = 7.1, 6.4 Hz, 1H). **^{13}C NMR (101 MHz, DMSO) δ** 161.6, 160.9, 149.6, 149.2, 143.0, 141.1, 140.2, 136.8, 134.5, 134.2 (2C), 132.7, 128.4, 126.2 (2C), 124.0, 121.4, 108.4, 105.1. **HRMS**: (APCI+) $[M+H]^+$ calc. for $C_{20}H_{13}Cl_2O_3N_4$, 427.0365, observed, 427.03592 .

Scheme S2: Synthesis towards target compound 7

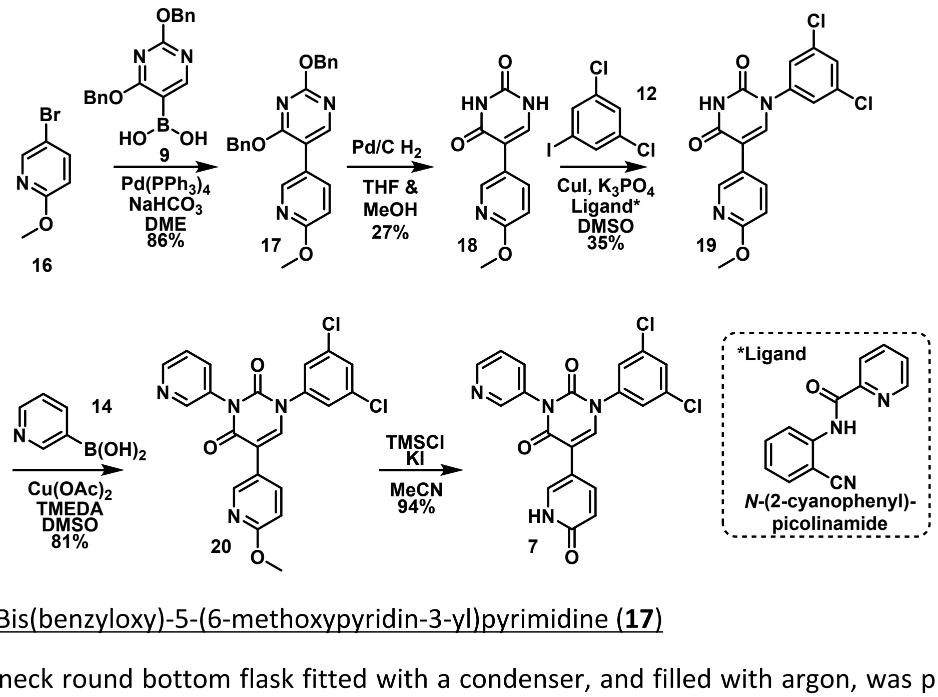

Synthesis of 2,4-Bis(benzyloxy)-5-(6-methoxypyridin-3-yl)pyrimidine (**17**)

Into a 500 mL 3 neck round bottom flask fitted with a condenser, and filled with argon, was placed 5-bromo-2-methoxypyridine **16** (4.50 g, 23.9 mmol) followed by DME (200 mL), thus forming a clear solution. To this was added (2,4-dibenzyloxypyrimidin-5-yl)boronic acid **9** (9.70 g, 28.9 mmol) which quickly dissolved and then

saturated sodium bicarbonate solution was added (150 mL), resulting in an immediate white precipitate forming. The solution was then degassed for 10 minutes by bubbling argon into the mixture and then tetrakis(triphenylphosphine)palladium(0) (5.50 g, 4.78 mmol) was added in one portion. The reaction mixture was heated to reflux under argon resulting in most of the white precipitate solubilising, and thus forming a yellow solution. The reaction was left to proceed for 6 h at reflux and the colour changed to a darker orange colour. After this time, it was cooled and poured into a separating funnel and diluted with EtOAc (400 mL) and water (300 mL). After vigorously shaking, the layers were allowed to separate, and the organic layer was collected. The aqueous layer was extracted twice more with EtOAc (2 x 200 mL). The organic fractions were combined and dried over anhydrous magnesium sulfate and then filtered. After concentrating to ca 300 mL, this organic phase was pulled through a filter bed of silica gel. The silica bed was washed with ethyl acetate. The organic solution was then concentrated *in vacuo* and the crude material purified by column chromatography (EtOAc/Hex) to afford 2,4-bis(benzyloxy)-5-(6-methoxypyridin-3-yl)pyrimidine **17** (8.24 g, 20.6 mmol, 86%) as a white solid.

Rf = 0.50 (30% EtOAc/Hexane). **^{1}H NMR (600 MHz, DMSO) δ** 8.45 (s, 1H), 8.38 – 8.33 (m, 1H), 7.90 (dd, *J* = 8.5, 2.5 Hz, 1H), 7.50 – 7.45 (m, 2H), 7.44 – 7.29 (m, 8H), 6.87 (d, *J* = 8.5 Hz, 1H), 5.47 (s, 2H), 5.44 (s, 2H), 3.86 (s, 3H). **^{13}C NMR (151 MHz, DMSO) δ** 167.0, 163.4, 162.9, 157.9, 146.2, 139.4, 136.6, 136.1, 128.5 (2C), 128.4 (2C), 128.0, 128.0 (2C), 128.0, 127.9 (2C), 122.1, 112.4, 110.1, 68.6, 68.1, 53.2. **HRMS**: (APCI+) $[M+H]^+$ calc. for $C_{24}H_{22}O_3N_3$, 400.1661, observed, 400.16515.

Synthesis of 5-(6-Methoxypyridin-3-yl)pyrimidine-2,4(1*H*,3*H*)-dione (**18**)

Into a 2 neck 500 mL round bottom flask containing ethanol (150 mL) and THF (150 mL) was placed 2,4-bis(benzyloxy)-5-(6-methoxypyridin-3-yl)pyrimidine **17** (8.20 g, 20.5 mmol) thus forming a clear solution, which was blanketed with argon. To this was then added 10% palladium on carbon (0.218 g, 2.05 mmol) in one portion. A balloon containing hydrogen was fitted to the flask and the flask was purged somewhat with hydrogen. The reaction left to proceed for 18 h at room temperature resulting in a white precipitate forming. The solids (precipitate and catalyst) were removed by filtration and the filter cake was washed with DMSO in an attempt to recover product. The DMSO washings, were combined and cooled overnight, resulting in precipitation of the product. This was filtered once again and the filter cake washed with MeOH/DCM to remove residual DMSO. Finally, the original filtrate was concentrated in vacuo and all product solids combined to afford 5-(6-methoxypyridin-3-yl)pyrimidine-2,4(1*H*,3*H*)-dione **18** (1.25 g, 5.7 mmol, 27%) as a white solid.

^{1}H NMR (600 MHz, DMSO) δ 11.28 (br s, 1H), 11.19 (br s, 1H), 8.30 – 8.27 (m, 1H), 7.90 (dd, *J* = 8.5, 2.5 Hz, 1H), 7.66 (s, 1H), 6.81 (d, *J* = 8.5 Hz, 1H), 3.85 (s, 3H). **^{13}C NMR (151 MHz, DMSO) δ** 163.3, 162.5, 151.0, 145.5, 139.2, 139.0, 122.7, 109.6, 109.2, 53.1. **HRMS**: (APCI+) $[M+H]^+$ calc. for $C_{10}H_{10}O_3N_3$, 220.0722, observed, 220.07129.

Synthesis of 1-(3-(Benzyloxy)-5-chlorophenyl)-5-(6-methoxypyridin-3-yl)pyrimidine-2,4(1*H*,3*H*)-dione (**19**)

A Schlenk tube was charged with 5-(6-methoxypyridin-3-yl)pyrimidine-2,4(1*H*,3*H*)-dione **18** (400 mg, 1.83 mmol), followed by 1,3-dichloro-5-iodobenzene **12** (500 mg, 1.83 mmol), K_3PO_4 (818 mg, 3.85 mmol), CuI (35 mg, 0.18 mmol) and *N*-(2-cyanophenyl)picolinamide (50 mg, 0.22 mmol). The vessel was evacuated and back filled with argon. DMSO (13 mL) was added and the reaction mixture was degassed for 15 min by bubbling argon into the solution. The reaction was stirred overnight at 60 °C. Once cooled, the reaction solution was diluted with EtOAc and washed with water. The aqueous layer was then extracted with EtOAc (x3). The combined organic layers were then washed with $Cu(OAc)_2$.H2O (x2), 15% NH_4Cl (x2), brine and dried over $MgSO_4$. After filtration and removal of solvent in vacuo, the crude material was purified using column chromatography (1-5% MeOH/DCM) to afford 1-(3,5-dichlorophenyl)-5-(6-methoxypyridin-3-yl)pyrimidine-2,4(1*H*,3*H*)-dione **19** (230 mg, 0.632 mmol) as an

impure white solid. Due to difficulties with in purifying this compound, it was progressed to the next step without further characterisation.

Synthesis of 1-(3,5-Dichlorophenyl)-5-(6-methoxypyridin-3-yl)-3-(pyridin-3-yl)pyrimidine-2,4(1*H*,3*H*)-dione (**20**)

To a round-bottomed flask was added 1-(3,5-dichlorophenyl)-5-(6-methoxypyridin-3-yl)pyrimidine-2,4(1*H*,3*H*)-dione **19** (230 mg, 0.632 mmol), followed by 3-pyridinyl boronic acid **14** (777 mg, 6.32 mmol) and DMSO (5 mL). Copper(II) acetate (230 mg, 1.27 mmol) was then added to the reaction solution followed by TMEDA (0.95 mL, 6.3 mmol). The reaction mixture was stirred at 60 °C open to the atmosphere and monitored using LCMS. Once completed, EtOAc was added and the solution was washed with H_2O (x2), brine and dried over $MgSO_4$. Filtration was then followed by *in vacuo* removal of solvent and purification by column chromatography (EtOAc/Hex) to afford 1-(3,5-dichlorophenyl)-5-(6-methoxypyridin-3-yl)-3-(pyridin-3-yl)pyrimidine-2,4(1*H*,3*H*)-dione **20** as a white solid (225 mg, 0.551 mmol, 81%).

Rf = 0.42 (80% EtOAc/Hexane).**^{1}H NMR (600 MHz, DMSO) δ** 8.63 (dd, *J* = 4.8, 1.5 Hz, 1H), 8.59 (dd, *J* = 2.4, 0.7 Hz, 1H), 8.44 (dd, *J* = 2.5, 0.7 Hz, 1H), 8.27 (s, 1H), 7.96 (dd, *J* = 8.7, 2.5 Hz, 1H), 7.86 (ddd, *J* = 8.1, 2.4, 1.6 Hz, 1H), 7.78 (d, *J* = 1.9 Hz, 2H), 7.76 (t, *J* = 1.9 Hz, 1H), 7.58 (ddd, *J* = 8.1, 4.8, 0.7 Hz, 1H), 6.87 (dd, *J* = 8.7, 0.7 Hz, 1H), 3.87 (s, 3H). **^{13}C NMR (151 MHz, DMSO) δ** 163.0, 161.7, 149.8, 149.5, 149.2, 146.1, 141.2, 140.8, 139.2, 136.7, 134.0 (2C), 132.5, 128.3, 126.4 (2C), 124.0, 122.0, 110.6, 109.8, 53.3. **HRMS**: (APCI+) $[M+H]^+$ calc. for $C_{21}H_{15}Cl_2O_3N_4$, 441.0521, observed, 441.05126.

Synthesis of 1-(3,5-Dichlorophenyl)-5-(6-oxo-1,6-dihydropyridin-3-yl)-3-(pyridin-3-yl)pyrimidine-2,4(1*H*,3*H*)-dione (**7**)

Reaction carried out according to G*eneral procedure-C*: Using 1-(3,5-dichlorophenyl)-5-(6-methoxypyridin-3-yl)-3-(pyridin-3-yl)pyrimidine-2,4(1H,3H)-dione **20** (200 mg, 0.455 mmol), NaI (170 mg, 1.14 mmol), TMSCl (123 mg, 1.14 mmol), acetonitrile (10 mL). Afforded 1-(3,5-dichlorophenyl)-5-(6-oxo-1,6-dihydropyridin-3-yl)-3-(pyridin-3-yl)pyrimidine-2,4(1*H*,3*H*)-dione **7** as an off-white solid (181 mg, 0.425 mmol, 94%).

Rf = 0.50 (10% MeOH/DCM). **^{1}H NMR (400 MHz, DMSO) δ** 11.80 (s, 1H), 8.62 (dd, *J* = 4.8, 1.5 Hz, 1H), 8.56 (dd, *J* = 2.5, 0.8 Hz, 1H), 8.19 (s, 1H), 7.84 (ddd, *J* = 8.1, 2.5, 1.6 Hz, 1H), 7.79 (dd, *J* = 2.7, 0.8 Hz, 1H), 7.78 – 7.74 (m, 3H), 7.73 (d, *J* = 2.7 Hz, 1H), 7.57 (ddd, *J* = 8.1, 4.8, 0.8 Hz, 1H), 6.37 (dd, *J* = 9.6, 0.7 Hz, 1H). **^{13}C NMR (101 MHz, DMSO) δ** 161.7, 161.7, 149.7, 149.6, 149.3, 141.1, 140.8, 140.1, 136.8, 134.4, 134.1 (2C), 132.5, 128.3, 126.5 (2C), 124.1, 119.3, 110.3, 109.7. **HRMS**: (APCI+) $[M+H]^+$ calc. for $C_{20}H_{13}Cl_2O_3N_4$, 427.0365, observed, 427.03580.

Synthesis of 1-(3-Benzyloxy-5-chloro-phenyl)-5-(2-methoxy-3-pyridyl)pyrimidine-2,4-dione (**22**)

Into a two neck round bottom flask containing argon was placed DMSO (40 mL) followed by 5-(2-methoxy-3-pyridyl)-1H-pyrimidine-2,4-dione **11** (2.00 g, 9.12 mmol), 1-benzyloxy-3-chloro-5-iodo-benzene **21** (3.77 g, 11.0 mmol), *N*-(2-cyanophenyl)picolinamide (Ligand*) (0.24 g, 1.1 mmol) and potassium phosphate (4.07 g, 19.2 mmol), thus forming a brown suspension. This mixture was degassed by bubbling argon into the solvent for 10 min and then copper(i) iodide (0.17 g, 0.91 mmol) was added against a flow of argon. The reaction mixture was heated to 60 °C and the colour of the mixture turned to very dark green. The reaction was left to proceed under argon at this temperature for three days. The reaction mixture was then diluted with EtOAc (200 mL) and water (500 mL). Upon mixing a suspension formed which was broken by pulling the entire mixture through a pad of celite. The organic layer was separated, and the aqueous layer extracted twice with EtOAc (2 x 200 mL). The combined organic fractions were then dried over anhydrous magnesium sulfate and filtered. The solvent was removed *in vacuo* and the crude material purified by column chromatography (EtOAc/Hex) to afford 1-(3-

benzyloxy-5-chloro-phenyl)-5-(2-methoxy-3-pyridyl)pyrimidine-2,4-dione **22** (1.84 g, 4.22 mmol, 46% yield) as a white solid.

^{1}H NMR (400 MHz, DMSO) δ 11.69 (s, 1H), 8.18 – 8.12 (m, 1H), 7.88 (s, 1H), 7.77 – 7.69 (m, 1H), 7.49 – 7.31 (m, 6H), 7.29 – 7.18 (m, 3H), 7.07 – 6.99 (m, 1H), 5.17 (s, 2H), 3.83 (s, 3H).

Synthesis of 1-(3-Benzyloxy-5-chloro-phenyl)-5-(2-methoxy-3-pyridyl)-3-(3-pyridyl)pyrimidine-2,4-dione (**23**)

Into a 500 mL one neck round bottom flask containing 1-(3-benzyloxy-5-chloro-phenyl)-5-(2-methoxy-3-pyridyl)pyrimidine-2,4-dione **22** (1.84 g, 4.22 mmol) was added 3-pyridylboronic acid **14** (1.56 g, 12.7 mmol), copper(II) acetate (1.15 g, 6.33 mmol), DMSO (20 mL) and finally TMEDA (1.27 mL, 8.44 mmol), forming a deep blue solution. This was stirred open to the atmosphere at 60 °C for 18 h after which analysis by LCMS indicated complete conversion of the starting material to the desired product. The reaction mixture was diluted with ethyl acetate (200 mL) and water (300 mL) and mixed vigorously. After separating the phases, the aqueous phase was extracted twice with EtOAc (2 x 200 mL) and the combined organic fractions were dried over anhydrous magnesium sulfate, filtered and concentrated *in vacuo*. The crude material was purified by column chromatography (EtOAc/Hex) affording 1-(3-benzyloxy-5-chloro-phenyl)-5-(2-methoxy-3-pyridyl)-3-(3-pyridyl)pyrimidine-2,4-dione **23** (1.94 g, 3.78 mmol, 90% yield) as a white solid.

^{1}H NMR (400 MHz, DMSO) δ 8.62 (dd, *J* = 4.8, 1.6 Hz, 1H), 8.59 (dd, *J* = 2.5, 0.8 Hz, 1H), 8.17 (dd, *J* = 5.0, 1.9 Hz, 1H), 8.12 (s, 1H), 7.86 (ddd, *J* = 8.1, 2.5, 1.6 Hz, 1H), 7.80 (dd, *J* = 7.3, 1.9 Hz, 1H), 7.57 (ddd, *J* = 8.1, 4.8, 0.8 Hz, 1H), 7.49 – 7.32 (m, 6H), 7.29 (t, *J* = 2.1 Hz, 1H), 7.24 (t, *J* = 2.1 Hz, 1H), 7.06 (dd, *J* = 7.4, 5.0 Hz, 1H), 5.18 (s, 2H), 3.85 (s, 3H).).

Scheme S3: Synthesis towards target compounds 35–45

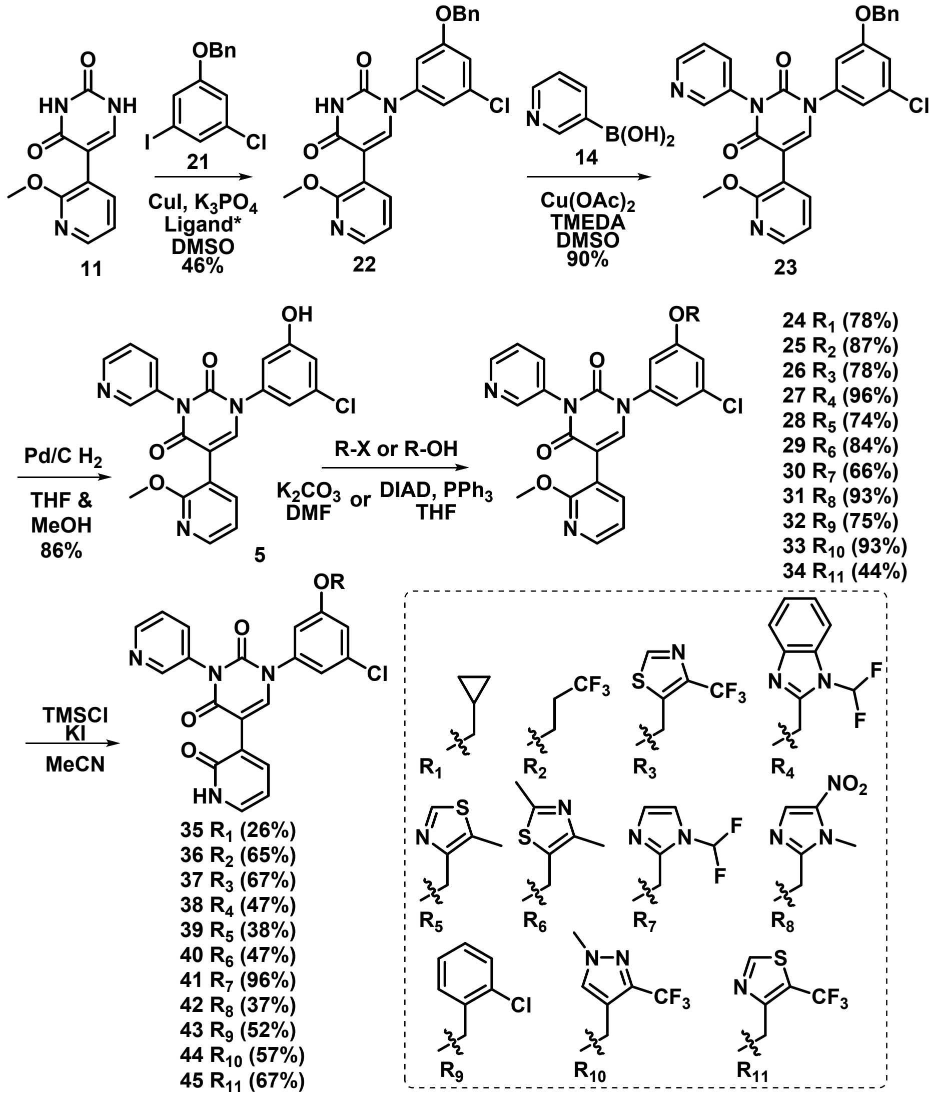

Synthesis of 1-(3-Chloro-5-hydroxy-phenyl)-5-(2-methoxy-3-pyridyl)-3-(3-pyridyl)pyrimidine-2,4-dione (**5**)

Into two neck round bottom flask containing argon was placed 1-(3-benzyloxy-5-chloro-phenyl)-5-(2-methoxy-3-pyridyl)-3-(3-pyridyl)pyrimidine-2,4-dione **23** (1.94 g, 3.78 mmol) followed by methanol (100 mL) and THF (100 mL). With heating, the starting material solubilised and remained in solution after cooling. To this was added Pd/C (0.40 g, 0.38 mmol) and a balloon filled with hydrogen was fitted to the flask. The atmosphere in the flask was purged with hydrogen gas and the reaction was left to proceed at 40 °C. After three hours, analysis by LCMS revealed that all of the starting material had been converted to the desired product and so the reaction mixture was filtered through celite and concentrated *in vacuo* to afford 1-(3-chloro-5-hydroxy-phenyl)-5-(2-methoxy-3-pyridyl)-3-(3-pyridyl)pyrimidine-2,4-dione **5** (1.38 g, 3.26 mmol, 86 % yield) as a white solid which was used without further purification.

^{1}H NMR (400 MHz, DMSO) δ 10.38 (s, 1H), 8.64 – 8.56 (m, 2H), 8.20 – 8.11 (m, 1H), 8.08 (s, 1H), 7.86 (m, 1H), 7.82 – 7.74 (m, 1H), 7.60 – 7.52 (m, 1H), 7.14 – 7.01 (m, 2H), 6.98 – 6.87 (m, 2H), 3.85 (s, 3H).

Synthesis of 1-[3-Chloro-5-(cyclopropylmethoxy)phenyl]-5-(2-methoxy-3-pyridyl)-3-(3-pyridyl)pyrimidine-2,4-dione (**24**)

Carried out according to *General procedure A* using 1-(3-chloro-5-hydroxy-phenyl)-5-(2-methoxy-3-pyridyl)-3-(3-pyridyl)pyrimidine-2,4-dione **5** (400 mg, 0.946 mmol) and bromomethylcyclopropane (0.11 mL, 1.1 mmol) afforded 1-[3-chloro-5-(cyclopropylmethoxy)phenyl]-5-(2-methoxy-3-pyridyl)-3-(3-pyridyl)pyrimidine-2,4-dione **24** (352 mg, 0.738 mmol, 78% yield).

^{1}H NMR (400 MHz, $CDCl_3$) δ 8.69 – 8.61 (m, 2H), 8.16 (dd, *J* = 4.9, 1.9 Hz, 1H), 7.82 (dd, *J* = 7.4, 1.9 Hz, 1H), 7.79 – 7.75 (m, 2H), 7.51 (dd, *J* = 8.2, 4.9 Hz, 1H), 7.04 (t, *J* = 1.8 Hz, 1H), 7.02 – 6.88 (m, 3H), 4.11 (q, *J* = 7.1 Hz, 1H), 3.97 (s, 3H), 3.82 (d, *J* = 6.9 Hz, 2H), 0.70 – 0.59 (m, 2H), 0.38 – 0.30 (m, 2H).

Synthesis of 1-[3-Chloro-5-(3,3,3-trifluoropropoxy)phenyl]-5-(2-methoxy-3-pyridyl)-3-(3-pyridyl)pyrimidine-2,4-dione (**25**)

Carried out according to *General procedure B*: Using 1-(3-chloro-5-hydroxy-phenyl)-5-(2-methoxy-3-pyridyl)-3-(3-pyridyl)pyrimidine-2,4-dione **5** (100 mg, 0.237 mmol) and 3,3,3-trifluoropropan-1-ol (37 mg, 0.331 mmol) afforded 1-[3-chloro-5-(3,3,3-trifluoropropoxy)phenyl]-5-(2-methoxy-3-pyridyl)-3-(3-pyridyl)pyrimidine-2,4-dione **25** (107 mg, 0.206 mmol, 87% yield) as a white solid.

^{1}H NMR (400 MHz, DMSO) δ 8.64 – 8.55 (m, 2H), 8.20 – 8.10 (m, 2H), 7.89 – 7.75 (m, 2H), 7.57 (dd, *J* = 8.2, 4.8 Hz, 1H), 7.38 – 7.00 (m, 4H), 4.29 (t, *J* = 5.8 Hz, 2H), 3.84 (s, 3H), 2.81 (qt, *J* = 11.4, 5.8 Hz, 2H).

Synthesis of 1-[3-Chloro-5-[[4-(trifluoromethyl)thiazol-5-yl]methoxy]phenyl]-5-(2-methoxy-3-pyridyl)-3-(3-pyridyl)pyrimidine-2,4-dione (**26**)

Carried out according to *General procedure B*: Using 1-(3-chloro-5-hydroxy-phenyl)-5-(2-methoxy-3-pyridyl)-3-(3-pyridyl)pyrimidine-2,4-dione **5** (172 mg, 0.407 mmol) and [4-(trifluoromethyl)thiazol-5-yl]methanol (78.8 mg, 0.430 mmol) **55** (see Scheme 5a for synthesis) afforded 1-[3-chloro-5-[[4-(trifluoromethyl)thiazol-5-yl]methoxy]phenyl]-5-(2-methoxy-3-pyridyl)-3-(3-pyridyl)pyrimidine-2,4-dione **26** (152 mg, 0.259 mmol, 78% yield) as a white solid.

^{1}H NMR (500 MHz, $CDCl_3$) δ 8.83 (s, 1H), 8.65 (dd, *J* = 4.9, 1.5 Hz, 1H), 8.62 – 8.57 (m, 1H), 8.16 – 8.13 (m, 1H), 7.83 – 7.80 (m, 1H), 7.80 – 7.79 (m, 1H), 7.71 – 7.66 (m, 1H), 7.46 – 7.42 (m, 1H), 7.18 – 7.15 (m, 1H), 7.07 – 7.03 (m, 2H), 6.97 – 6.92 (m, 1H), 5.42 – 5.40 (m, 2H), 3.97 (s, 3H). **^{13}C NMR (101 MHz, $CDCl_3$) δ** 213.9, 201.8, 161.4, 160.8, 158.5, 153.8, 153.7, 149.9, 149.8, 149.7, 149.5, 149.4, 146.9, 142.5, 140.5, 134.0, 136.6, 136.2, 136.1, 131.7, 123.9, 120.4, 116.8, 115.8, 114.5, 112.2, 110.4, 62.5, 54.0, 53.9. **HRMS** (APCI) m/z calculated for $C_{26}H_{18}O_4N_5{}^{35}ClF_3{}^{32}S$ $[M+H]^+$ 588.0715 found 588.0715.

Synthesis of 1-[3-Chloro-5-[[1-(difluoromethyl)benzimidazol-2-yl]methoxy]phenyl]-5-(2-methoxy-3-pyridyl)-3-(3-pyridyl)pyrimidine-2,4-dione (**27**)

Carried out according to *General procedure A* using 1-(3-chloro-5-hydroxy-phenyl)-5-(2-methoxy-3-pyridyl)-3-(3-pyridyl)pyrimidine-2,4-dione **5** (300 mg, 0.710 mmol) and 2-(chloromethyl)-1-(difluoromethyl)benzimidazole hydrochloride (233 mg, 0.922 mmol) afforded 1-[3-chloro-5-[[1-(difluoromethyl)benzimidazol-2-yl]methoxy]phenyl]-5-(2-methoxy-3-pyridyl)-3-(3-pyridyl)pyrimidine-2,4-dione **27** (411 mg, 0.682 mmol, 96% yield).

^{1}H NMR (600 MHz, DMSO) δ 8.61 (dt, *J* = 4.9, 1.4 Hz, 1H), 8.59 – 8.58 (m, 1H), 8.19 – 8.14 (m, 2H), 8.10 (s, 1H), 7.88 – 7.84 (m, 1H), 7.82 – 7.73 (m, 3H), 7.59 – 7.54 (m, 1H), 7.47 – 7.42 (m, 1H), 7.41 – 7.33 (m, 4H), 7.06 (ddd, *J* = 7.4, 4.9, 1.1 Hz, 1H), 5.62 (s, 2H), 3.84 (s, 3H).

Synthesis of 1-(3-Chloro-5-[(5-methylthiazol-4-yl)methoxy]phenyl]-5-(2-methoxy-3-pyridyl)-3-(3-pyridyl) pyrimidine-2,4-dione (**28**)

Carried out according to *General procedure* A: Using 1-(3-chloro-5-hydroxy-phenyl)-5-(2-methoxy-3-pyridyl)-3-(3-pyridyl)pyrimidine-2,4-dione **5** (400 mg, 0.946 mmol) and 4-(chloromethyl)-5-methyl-thiazole hydrochloride (226 mg, 1.23 mmol) afforded 1-[3-chloro-5-[(5-methylthiazol-4-yl)methoxy]phenyl]-5-(2-methoxy-3-pyridyl)-3-(3-pyridyl)pyrimidine-2,4-dione **28** (372 mg, 0.697 mmol, 74 % yield) as an off-white solid.

^{1}H NMR (400 MHz, $CDCl_3$) δ 8.67 – 8.62 (m, 2H), 8.58 (s, 1H), 8.15 (dd, *J* = 5.0, 1.9 Hz, 1H), 7.81 (dd, *J* = 7.4, 1.9 Hz, 1H), 7.79 – 7.74 (m, 2H), 7.50 (ddd, *J* = 8.2, 4.9, 0.8 Hz, 1H), 7.16 – 7.03 (m, 3H), 6.95 (dd, *J* = 7.5, 5.0 Hz, 1H), 5.17 (s, 2H), 3.96 (s, 3H), 2.51 (s, 3H).

Synthesis of 1-[3-Chloro-5-[(2,4-dimethylthiazol-5-yl)methoxy]phenyl]-5-(2-methoxy-3-pyridyl)-3-(3-pyridyl) pyrimidine-2,4-dione (**29**)

Carried out according to *General procedure A* using 1-(3-chloro-5-hydroxy-phenyl)-5-(2-methoxy-3-pyridyl)-3-(3-pyridyl)pyrimidine-2,4-dione **5** (300 mg, 0.710 mmol) and 5-(chloromethyl)-2,4-dimethyl-thiazole hydrochloride (169 mg, 0.851 mmol) afforded 1-[3-chloro-5-[(2,4-dimethylthiazol-5-yl)methoxy]phenyl]-5-(2-methoxy-3-pyridyl)-3-(3-pyridyl)pyrimidine-2,4-dione **29** (328 mg, 0.599 mmol, 84% yield) as a white solid.

^{1}H NMR (400 MHz, DMSO) δ 8.76 – 8.68 (m, 2H), 8.19 – 8.16 (m, 1H), 8.14 (s, 1H), 8.06 (s, 1H), 7.82 – 7.76 (m, 1H), 7.76 – 7.69 (m, 1H), 7.39 – 7.33 (m, 1H), 7.29 – 7.23 (m, 2H), 7.10 – 7.04 (m, 1H), 5.31 (s, 2H), 3.85 (s, 3H), 2.60 (s, 3H), 2.33 (s, 3H).

Synthesis of 1-[3-Chloro-5-[[1-(difluoromethyl)imidazol-2-yl]methoxy]phenyl]-5-(2-methoxy-3-pyridyl)-3-(3-pyridyl)pyrimidine-2,4-dione (**30**)

Carried out according to *General procedure A*: Using 1-(3-chloro-5-hydroxy-phenyl)-5-(2-methoxy-3-pyridyl)-3-(3-pyridyl)pyrimidine-2,4-dione **5** (172 mg, 0.407 mmol) and 2-(chloromethyl)-1-(difluoromethyl)imidazole hydrochloride (246 mg, 1.21 mmol) afforded 1-[3-chloro-5-[[1-(difluoromethyl)imidazol-2-yl]methoxy]phenyl]-5-(2-methoxy-3-pyridyl)-3-(3-pyridyl)pyrimidine-2,4-dione **30** (149 mg, 0.270 mmol, 66% yield) as a yellow solid.

^{1}H NMR (500 MHz, CD_3OD) δ 8.60 (dd, *J* = 4.9, 1.5 Hz, 1H), 8.59 (dd, *J* = 2.4, 0.8 Hz, 1H), 8.14 (dd, *J* = 5.0, 1.9 Hz, 1H), 7.95 (s, 1H), 7.91 (ddd, *J* = 8.2, 2.5, 1.5 Hz, 1H), 7.81 (dd, *J* = 7.4, 1.9 Hz, 1H), 7.70 (t, *J* = 59.2 Hz, 1H), 7.61 (ddd, *J* = 8.2, 4.9, 0.8 Hz, 1H), 7.56 (d, *J* = 1.7 Hz, 1H), 7.30 (t, *J* = 1.8 Hz, 1H), 7.12 (d, *J* = 1.7 Hz, 1H), 7.01 (dd, *J* = 7.4, 5.0 Hz, 1H), 5.35 (s, 2H), 3.93 (s, 3H) ppm; **^{19}F NMR (471 MHz, CD_3OD)** δ -95.27 (d, *J* = 59.0 Hz, 2F) ppm;**^{13}C NMR (101 MHz, CD_3OD)** δ 163.5, 162.9, 160.2, 152.0, 150.4, 150.0, 147.7, 144.8, 143.9, 142.2, 141.6, 138.9, 136.5, 134.4, 129.8, 125.7, 121.8, 118.8, 117.9, 117.2, 117.1, 113.8, 111.9, 109.9 (t, *J* = 250.5 Hz), 63.5, 54.2 ppm; **HRMS** (APCI) m/z: $[M + H]^+$ Calcd for $C_{26}H_{20}O_4ClF_2N_6$ 553.1197; Found 553.1194

Synthesis of 1-(3-Chloro-5-((1-methyl-5-nitro-1H-imidazol-2-yl)methoxy)phenyl)-5-(2-methoxypyridin-3-yl)-3-(pyridin-3-yl)pyrimidine-2,4-dione (**31**)

Carried out according to *General procedure A*: Using 1-(3-chloro-5-hydroxy-phenyl)-5-(2-methoxy-3-pyridyl)-3-(3-pyridyl)pyrimidine-2,4-dione **5** (250 mg, 0.591 mmol) and 2-(chloromethyl)-1-methyl-5-nitro-1H-imidazole hydrochloride (188 mg, 0.887 mmol) afforded 1-(3-chloro-5-((1-methyl-5-nitro-1H-imidazol-2-yl)methoxy)phenyl)-5-(2-methoxypyridin-3-yl)-3-(pyridin-3-yl)pyrimidine-2,4(1*H*,3*H*)-dione **31** (308 mg, 0.450 mmol, 93% yield) as a light brown solid.

Rf = 0.43 (5% MeOH/DCM). **^{1}H NMR (600 MHz, $CDCl_3$) δ** 8.66 (dd, *J* = 4.9, 1.5 Hz, 1H), 8.61 (d, *J* = 2.5 Hz, 1H), 8.16 (dd, *J* = 5.0, 1.9 Hz, 1H), 7.95 (s, 1H), 7.82 (dd, *J* = 7.4, 1.9 Hz, 1H), 7.76 (s, 1H), 7.72 (ddd, *J* = 8.1, 2.5, 1.5 Hz, 1H), 7.48 (ddd, *J* = 8.2, 4.9, 0.8 Hz, 1H), 7.16 – 7.08 (m, 3H), 6.96 (dd, *J* = 7.4, 4.9 Hz, 1H), 5.23 (s, 2H), 4.03 (s, 3H), 3.97 (s, 3H). **^{13}C NMR (151 MHz, $CDCl_3$) δ** 161.4, 160.9, 158.5, 150.0, 149.4, 149.2, 147.0, 146.3, 142.6, 140.6, 140.1, 140.0, 136.8, 136.3, 132.0, 131.8, 124.1, 120.5, 116.9, 116.2, 114.6, 112.2, 110.6, 63.2, 54.1, 34.0. **HRMS**: (APCI+) $[M+H]^+$ calc. for $C_{26}H_{21}ClO_6N_7$, 562.1242, observed, 562.1239.

Synthesis of 1-[3-Chloro-5-[(2-Chlorophenyl)methoxy]phenyl]-5-(2-methoxy-3-pyridyl)-3-(3-pyridyl)pyrimidine-2,4-dione (**32**)

Carried out according to *General procedure A* using 1-(3-chloro-5-hydroxy-phenyl)-5-(2-methoxy-3-pyridyl)-3-(3-pyridyl)pyrimidine-2,4-dione **5** (400 mg, 0.946 mmol) and 1-chloro-2-(chloromethyl)benzene (0.14 mL, 1.1 mmol) afforded 1-[3-chloro-5-[(2-chlorophenyl)methoxy]phenyl]-5-(2-methoxy-3-pyridyl)-3-(3-pyridyl)pyrimidine-2,4-dione **32** (390 mg, 0.713 mmol, 75 % yield) as an off-white solid.

^{1}H NMR (400 MHz, $CDCl_3$) δ 8.69 – 8.61 (m, 2H), 8.16 (dd, *J* = 5.0, 1.9 Hz, 1H), 7.82 (dd, *J* = 7.5, 1.9 Hz, 1H), 7.79 (s, 1H), 7.76 (ddd, *J* = 8.1, 2.5, 1.5 Hz, 1H), 7.55 – 7.46 (m, 2H), 7.45 – 7.36 (m, 1H), 7.34 – 7.27 (m, 2H), 7.12 (t, *J* = 1.8 Hz, 1H), 7.08 (t, *J* = 2.0 Hz, 1H), 7.04 (t, *J* = 2.1 Hz, 1H), 6.95 (dd, *J* = 7.4, 5.0 Hz, 1H), 5.17 (s, 2H), 3.97 (s, 3H).

Synthesis of 1-[3-Chloro-5-[[1-methyl-3-(trifluoromethyl)pyrazol-4-yl]methoxy]phenyl]-5-(2-methoxy-3-pyridyl)-3-(3-pyridyl)pyrimidine-2,4-dione (**33**)

Carried out according to *General procedure B*: Using 1-(3-chloro-5-hydroxy-phenyl)-5-(2-methoxy-3-pyridyl)-3-(3-pyridyl)pyrimidine-2,4-dione **5** (100 mg, 0.237 mmol) and [1-methyl-3-(trifluoromethyl)pyrazol-4-yl]methanol (59.6 mg, 0.331 mmol) **57** (see Scheme 5b for synthesis) afforded 1-[3-chloro-5-[[1-methyl-3-(trifluoromethyl)pyrazol-4-yl]methoxy]phenyl]-5-(2-methoxy-3-pyridyl)-3-(3-pyridyl)pyrimidine-2,4-dione **33** (134 mg, 0.229 mmol, 97 % yield) as a white solid.

^{1}H NMR (400 MHz, $CDCl_3$) δ 8.63 (dd, *J* = 4.8, 1.5 Hz, 1H), 8.60 – 8.57 (m, 1H), 8.14 (dd, *J* = 5.0, 1.9 Hz, 1H), 7.81 (dd, *J* = 7.4, 1.9 Hz, 1H), 7.78 (s, 1H), 7.68 (ddd, *J* = 8.1, 2.6, 1.5 Hz, 1H), 7.50 (s, 1H), 7.45 – 7.41 (m, 1H), 7.09 (t, *J* = 1.8 Hz, 1H), 7.02 – 6.96 (m, 1H), 6.94 (dd, *J* = 7.4, 5.0 Hz, 2H), 5.01 (s, 2H), 3.96 (s, 3H), 3.90 (s, 3H). **^{13}C NMR (101 MHz, $CDCl_3$)** δ 161.5, 160.9, 159.4, 150.0, 149.8, 149.7, 149.6, 149.5, 146.9, 146.8, 142.8, 140.4, 140.0, 136.3, 135.8, 132.1, 132.0, 131.9, 124.0, 123.9, 119.6, 119.6, 116.9, 116.9, 116.0, 115.2, 114.6, 112.1, 110.3, 60.8, 54.0, 54.0, 39.7.

Synthesis of 1-[3-Chloro-5-[[1-methyl-3-(trifluoromethyl)pyrazol-4-yl]methoxy]phenyl]-5-(2-methoxy-3-pyridyl)-3-(3-pyridyl)pyrimidine-2,4-dione (**34**)

Carried out according to *General procedure B*: Using 1-(3-chloro-5-hydroxy-phenyl)-5-(2-methoxy-3-pyridyl)-3-(3-pyridyl)pyrimidine-2,4-dione **5** (100 mg, 0.237 mmol) and [1-methyl-3-(trifluoromethyl)pyrazol-4-yl]methanol **57** (see Scheme 5b for synthesis) (59.6 mg, 0.331 mmol) afforded 1-[3-chloro-5-[[1-methyl-3-(trifluoromethyl)pyrazol-4-yl]methoxy]phenyl]-5-(2-methoxy-3-pyridyl)-3-(3-pyridyl)pyrimidine-2,4-dione **34** (54 mg, 0.10 mmol, 44% yield) as a white solid.

^{1}H NMR (400 MHz, $CDCl_3$) δ 8.63 (dd, *J* = 4.8, 1.5 Hz, 1H), 8.59 (dd, *J* = 2.6, 0.8 Hz, 1H), 8.14 (dd, *J* = 5.0, 1.9 Hz, 1H), 7.81 (dd, *J* = 7.5, 1.9 Hz, 1H), 7.78 (s, 1H), 7.68 (ddd, *J* = 8.1, 2.6, 1.5 Hz, 1H), 7.50 (s, 1H), 7.45 – 7.41 (m, 1H), 7.09 (t, *J* = 1.8 Hz, 1H), 7.00 (t, *J* = 2.0 Hz, 1H), 6.97 (t, *J* = 2.1 Hz, 1H), 6.94 (dd, *J* = 7.4, 5.0 Hz, 1H), 5.01 (s, 2H), 3.96 (s, 3H), 3.90 (s, 3H). **^{13}C NMR (101 MHz, $CDCl_3$)** δ 161.5, 160.9, 159.4, 150.0, 149.8, 149.7, 149.6, 149.5, 146.9,

146.8, 142.8, 140.4, 140.0, 136.3, 135.9, 132.2, 132.1, 132.0, 131.9, 128.7, 124.0, 123.9, 122.7, 120.1, 119.6, 119.6, 116.9, 116.8, 116.0, 115.2, 114.6, 112.1, 110.3, 60.8, 54.0, 54.0, 39.7. **HRMS** (APCI) m/z calculated for $C_{27}H_{21}O_4N_6{}^{35}ClF_3$ $[M+H]^+$ 585.1259 found 585.1257.

<u>Synthesis of 1-[3-Chloro-5-(cyclopropylmethoxy)phenyl]-5-(2-oxo-1H-pyridin-3-yl)-3-(3-pyridyl)pyrimidine-2,4-dione (**35**)</u>

Carried out according to *General procedure C*: Using 1-[3-chloro-5-(cyclopropylmethoxy)phenyl]-5-(2-methoxy-3-pyridyl)-3-(3-pyridyl)pyrimidine-2,4-dione **24** (352 mg, 0.738 mmol) afforded 1-[3-chloro-5-(cyclopropylmethoxy)phenyl]-5-(2-oxo-1H-pyridin-3-yl)-3-(3-pyridyl)pyrimidine-2,4-dione **35** (88 mg, 0.19 mmol, 26% yield) as a white solid.

^{1}H NMR (400 MHz, DMSO) δ 11.90 (s, 1H), 8.71 (s, 1H), 8.62 (dd, *J* = 4.8, 1.6 Hz, 1H), 8.59 – 8.52 (m, 1H), 8.04 (dd, *J* = 7.2, 2.1 Hz, 1H), 7.85 (ddd, *J* = 8.1, 2.5, 1.5 Hz, 1H), 7.57 (ddd, *J* = 8.1, 4.8, 0.8 Hz, 1H), 7.37 (dd, *J* = 6.4, 2.1 Hz, 1H), 7.26 (t, *J* = 1.8 Hz, 1H), 7.15 (p, *J* = 2.3 Hz, 2H), 6.30 (dd, *J* = 7.1, 6.4 Hz, 1H), 3.89 (d, *J* = 7.1 Hz, 2H), 1.30 – 1.13 (m, 1H), 0.64 – 0.51 (m, 2H), 0.37 – 0.29 (m, 2H). **^{13}C NMR (101 MHz, DMSO) δ** 162.1, 161.4, 160.2, 150.0, 150.0, 149.5, 143.9, 141.6, 140.4, 137.4, 134.7, 134.3, 133.2, 124.5, 121.8, 119.5, 115.3, 113.1, 108.3, 105.6, 73.5, 10.3, 3.6. **HRMS**: (APCI+) $[M+H]^+$ calc. for $C_{24}H_{20}ClN_4O_4$, 463.11676, observed, 463.11671.

<u>Synthesis of 1-[3-Chloro-5-(3,3,3-trifluoropropoxy)phenyl]-5-(2-oxo-1H-pyridin-3-yl)-3-(3-pyridyl)pyrimidine-2,4-dione (**36**)</u>

Carried out according to *General procedure C*: Using 1-[3-chloro-5-(3,3,3-trifluoropropoxy)phenyl]-5-(2-methoxy-3-pyridyl)-3-(3-pyridyl)pyrimidine-2,4-dione **25** (500 mg, 0.964 mmol) afforded 1-[3-chloro-5-(3,3,3-trifluoropropoxy)phenyl]-5-(2-oxo-1H-pyridin-3-yl)-3-(3-pyridyl)pyrimidine-2,4-dione **36** (318 mg, 0.630 mmol, 65% yield) as a white solid.

^{1}H NMR (400 MHz, DMSO) δ 11.90 (s, 1H), 8.70 (s, 1H), 8.61 (dd, *J* = 4.8, 1.5 Hz, 1H), 8.56 (d, *J* = 2.4 Hz, 1H), 8.03 (dd, *J* = 7.1, 2.1 Hz, 1H), 7.84 (ddd, *J* = 8.1, 2.5, 1.5 Hz, 1H), 7.61 – 7.53 (m, 1H), 7.40 – 7.34 (m, 1H), 7.32 (t, *J* = 1.8 Hz, 1H), 7.25 – 7.18 (m, 2H), 6.29 (t, *J* = 6.8 Hz, 1H), 4.29 (t, *J* = 5.9 Hz, 2H), 2.82 (qt, *J* = 11.4, 5.8 Hz, 2H). **^{13}C NMR (151 MHz, DMSO) δ** 161.66, 160.91, 158.94, 149.60, 149.49, 149.10, 143.39, 141.14, 139.97, 136.77, 134.28, 133.94, 132.72, 126.62 (q, $J_{C\text{-}F}$ = 277.0 Hz), 123.97, 121.35, 119.74, 114.78, 112.83, 107.91, 105.06, 61.85 (d, $J_{C\text{-}F}$ = 4.0 Hz), 32.61 (q, $J_{C\text{-}F}$ = 28.0 Hz). **HRMS**: (APCI+) $[M+H]^+$ calc. for $C_{23}H_{17}ClF_3N_4O_4$, 505.08849, observed, 505.08853.

<u>Synthesis of 1-(3-Chloro-5-((4-(trifluoromethyl)thiazol-5-yl)methoxy)phenyl)-5-(2-oxo-1,2-dihydropyridin-3-yl)-3-(pyridin-3-yl)pyrimidine-2,4-dione (**37**)</u>

Carried out according to *General procedure C*: Using 1-[3-chloro-5-[[4-(trifluoromethyl)thiazol-5-yl]methoxy]phenyl]-5-(2-methoxy-3-pyridyl)-3-(3-pyridyl)pyrimidine-2,4-dione **26** (152 mg, 0.259 mmol) afforded 1-(3-chloro-5-((4-(trifluoromethyl)thiazol-5-yl)methoxy)phenyl)-5-(2-oxo-1,2-dihydropyridin-3-yl)-3-(pyridin-3-yl)pyrimidine-2,4-dione **37** (100 mg, 0.174 mmol, 67% yield) as a white solid.

^{1}H NMR (600 MHz, DMSO) δ 11.90 (s, 1H), 9.27 (s, 1H), 8.72 – 8.70 (m, 1H), 8.63 – 8.60 (m, 1H), 8.58 – 8.56 (m, 1H), 8.05 – 8.00 (m, 1H), 7.86 – 7.81 (m, 1H), 7.57 (dd, *J* = 8.1, 4.7 Hz, 1H), 7.40 – 7.34 (m, 3H), 7.32 – 7.30 (m, 1H), 6.32 – 6.27 (m, 1H), 5.60 (s, 2H).**^{13}C NMR (151 MHz, DMSO**) δ 161.7, 160.9, 158.4, 156.4, 149.6, 149.5, 149.1, 143.3, 141.2, 134.0, 139.7, 139.5, 139.3, 139.0, 137.6, 137.6, 136.8, 134.3, 134.0, 132.7, 124.0, 123.8, 122.0, 121.3, 120.4, 120.2, 118.4, 115.1, 113.3, 108.0, 105.1, 61.7. **^{19}F NMR (565 MHz, DMSO**) δ -59.62. **HRMS** (APCI) m/z calculated for $C_{25}H_{16}O_4N_5{}^{35}ClF_3{}^{32}S$ $[M+H]^+$ 574.0558 found 574.0556.

Synthesis of 1-[3-Chloro-5-[[1-(difluoromethyl)benzimidazol-2-yl]methoxy]phenyl]-5-(2-oxo-1H-pyridin-3-yl)-3-(3-pyridyl)pyrimidine-2,4-dione (**38**)

Carried out according to *General procedure C*: Using 1-[3-chloro-5-[[1-(difluoromethyl)benzimidazol-2-yl]methoxy]phenyl]-5-(2-methoxy-3-pyridyl)-3-(3-pyridyl)pyrimidine-2,4-dione **27** (400 mg, 0.663 mmol) afforded 1-[3-chloro-5-[[1-(difluoromethyl)benzimidazol-2-yl]methoxy]phenyl]-5-(2-oxo-1H-pyridin-3-yl)-3-(3-pyridyl)pyrimidine-2,4-dione **38** (182 mg, 0.309 mmol, 47% yield) as a white solid.

^{1}H NMR (400 MHz, DMSO) δ 11.93 (s, 1H), 8.69 (s, 1H), 8.65 – 8.55 (m, 2H), 8.36 – 7.99 (m, 2H), 7.85 (ddd, *J* = 8.2, 1.8 Hz, 1H), 7.77 (dd, *J* = 14.2, 7.9 Hz, 2H), 7.57 (dd, *J* = 8.1, 4.8 Hz, 1H), 7.50 – 7.30 (m, 6H), 6.30 (t, *J* = 6.8 Hz, 1H), 5.62 (s, 2H). **^{13}C NMR (101 MHz, DMSO) δ** 161.7, 161.0, 158.5, 149.6, 149.5, 149.2, 147.5, 143.4, 141.9, 141.1, 140.1, 136.8, 134.4, 133.9, 132.8, 131.4, 125.1, 124.1, 124.0, 121.3, 120.4, 115.1, 113.4, 112.2, 109.6, 108.0, 105.1, 63.2, 18.6. **HRMS**: (APCI+) $[M+H]^+$ calc. for $C_{29}H_{20}ClF_2N_6O_4$, 589.11971, observed, 589.12008.

Synthesis of 1-[3-chloro-5-[(5-methylthiazol-4-yl)methoxy]phenyl]-5-(2-oxo-1H-pyridin-3-yl)-3-(3-pyridyl)pyrimidine-2,4-dione (**39**)

Carried out according to *General procedure C*: Using 1-[3-chloro-5-[(5-methylthiazol-4-yl)methoxy]phenyl]-5-(2-methoxy-3-pyridyl)-3-(3-pyridyl)pyrimidine-2,4-dione **28** (372 mg, 0.697 mmol) afforded 1-[3-chloro-5-[(5-methylthiazol-4-yl)methoxy]phenyl]-5-(2-oxo-1H-pyridin-3-yl)-3-(3-pyridyl)pyrimidine-2,4-dione **39** (137 mg, 0.264 mmol, 38 % yield) as an off white solid.

^{1}H NMR (400 MHz, DMSO) δ 11.92 (s, 1H), 8.90 (s, 1H), 8.71 (s, 1H), 8.61 (dd, *J* = 4.8, 1.6 Hz, 1H), 8.57 (dd, *J* = 2.5, 0.8 Hz, 1H), 8.03 (dd, *J* = 7.1, 2.1 Hz, 1H), 7.85 (ddd, *J* = 8.1, 2.5, 1.6 Hz, 1H), 7.57 (ddd, *J* = 8.1, 4.8, 0.8 Hz, 1H), 7.38 (dd, *J* = 6.4, 2.1 Hz, 1H), 7.32 (d, *J* = 2.0 Hz, 2H), 7.25 (t, *J* = 2.1 Hz, 1H), 6.37 – 6.25 (m, 1H), 5.22 (s, 2H), 2.50 (s, 3H). **^{13}C NMR (101 MHz, DMSO) δ** 161.7, 160.9, 159.5, 151.1, 149.6, 149.5, 149.1, 147.0, 143.4, 141.1, 140.0, 136.8, 134.3, 133.8, 133.3, 132.8, 124.0, 121.4, 119.5, 114.9, 113.1, 108.0, 105.1, 63.9, 10.7. **HRMS**: (APCI+) $[M+H]^+$ calc. for $C_{25}H_{19}ClN_5O_4S$, 520.08408, observed, 520.08437.

Synthesis of 1-[3-Chloro-5-[(2,4-dimethylthiazol-5-yl)methoxy]phenyl]-5-(2-oxo-1H-pyridin-3-yl)-3-(3-pyridyl)pyrimidine-2,4-dione (**40**)

Carried out according to *General procedure C*: Using 1-[3-chloro-5-[(2,4-dimethylthiazol-5-yl)methoxy]phenyl]-5-(2-methoxy-3-pyridyl)-3-(3-pyridyl)pyrimidine-2,4-dione **29** (328 mg, 0.599 mmol) afforded 1-[3-chloro-5-[(2,4-dimethylthiazol-5-yl)methoxy]phenyl]-5-(2-oxo-1*H*-pyridin-3-yl)-3-(3-pyridyl)pyrimidine-2,4-dione **40** (150 mg, 0.281 mmol, 47% yield) as a white solid.

^{1}H NMR (400 MHz, DMSO) δ 11.92 (s, 1H), 8.71 (s, 1H), 8.62 (dd, *J* = 4.8, 1.5 Hz, 1H), 8.57 (dd, *J* = 2.5, 0.8 Hz, 1H), 8.03 (dd, *J* = 7.2, 2.1 Hz, 1H), 7.85 (ddd, *J* = 8.1, 2.5, 1.6 Hz, 1H), 7.57 (ddd, *J* = 8.1, 4.8, 0.8 Hz, 1H), 7.38 (dd, *J* = 6.4, 2.1 Hz, 1H), 7.33 (t, *J* = 1.8 Hz, 1H), 7.28 (t, *J* = 2.1 Hz, 1H), 7.23 (t, *J* = 2.1 Hz, 1H), 6.30 (dd, *J* = 7.2, 6.4 Hz, 1H), 5.30 (s, 2H), 2.58 (s, 3H), 2.33 (s, 3H). **^{13}C NMR (101 MHz, DMSO) δ** 164.6, 161.7, 160.9, 158.9, 150.6, 149.6, 149.5, 149.2, 143.4, 141.1, 140.0, 136.8, 134.3, 133.9, 132.8, 124.9, 124.0, 121.3, 119.8, 115.1, 113.3, 108.0, 105.1, 62.2, 18.8, 14.8. **HRMS**: (APCI+) $[M+H]^+$ calc. for $C_{26}H_{21}ClN_5O_4S$, 534.09973, observed, 534.10008. **HRMS**: (APCI+) $[M+H]^+$ calc. for $C_{26}H_{21}ClN_5O_4S$, 534.09973, observed, 534.10008.

Synthesis of 1-[3-Chloro-5-[[1-(difluoromethyl)imidazol-2-yl]methoxy]phenyl]-5-(2-methoxy-3-pyridyl)-3-(3-pyridyl)pyrimidine-2,4-dione (**41**)

Carried out according to *General procedure C*: Using 1-[3-chloro-5-[[1-(difluoromethyl)imidazol-2-yl]methoxy]phenyl]-5-(2-methoxy-3-pyridyl)-3-(3-pyridyl)pyrimidine-2,4-dione **30** (128 mg, 0.232 mmol) afforded 1-[3-chloro-5-[[1-(difluoromethyl)imidazol-2-yl]methoxy]phenyl]-5-(2-oxo-1H-pyridin-3-yl)-3-(3-pyridyl)pyrimidine-2,4-dione **41** (120 mg, 0.223 mmol, 96% yield) as an off white solid.

^{1}H NMR (400 MHz, DMSO) δ 11.93 (s, 1H), 8.70 (s, 1H), 8.61 (dd, *J* = 4.8, 1.6 Hz, 1H), 8.57 (dd, *J* = 2.5, 0.7 Hz, 1H), 8.03 (dd, *J* = 7.1, 2.1 Hz, 1H), 7.93 (t, *J* = 58.8 Hz, 1H), 7.85 (ddd, *J* = 8.1, 2.5, 1.6 Hz, 1H), 7.69 (d, *J* = 1.6 Hz, 1H), 7.57 (ddd, *J* = 8.1, 4.8, 0.8 Hz, 1H), 7.38 (dd, *J* = 6.4, 2.1 Hz, 1H), 7.36 (t, *J* = 1.9 Hz, 1H), 7.33 (t, *J* = 2.1 Hz, 1H), 7.26 (t, *J* = 2.1 Hz, 1H), 7.13 (d, *J* = 1.6 Hz, 1H), 6.30 (dd, *J* = 7.1, 6.4 Hz, 1H), 5.37 (s, 2H) ppm; **^{19}F NMR (376 MHz, DMSO) δ** -92.49 (d, *J* = 58.6 Hz, 2F) ppm; **^{13}C NMR (101 MHz, DMSO) δ** 161.7, 161.0, 158.6, 149.6, 149.5, 149.1, 143.3, 142.0, 141.1, 140.0, 136.8, 134.4, 133.8, 132.7, 129.4, 124.0, 121.3, 120.2, 117.7, 115.0, 113.3, 108.4 (t, *J* = 248.4 Hz), 108.0, 105.1, 62.4 ppm; **HRMS** (APCI) m/z: $[M + H]^+$ Calcd for $C_{25}H_{18}O_4ClF_2N_6$ 539.1041; Found 539.1044.

<u>Synthesis of 1-(3-Chloro-5-((1-methyl-5-nitro-1H-imidazol-2-yl)methoxy)phenyl)-5-(2-oxo-1,2-dihydropyridin-3-yl)-3-(pyridin-3-yl)pyrimidine-2,4-dione (**42**)</u>

Carried out according to *General procedure C*: Using 1-(3-chloro-5-((1-methyl-5-nitro-1H-imidazol-2-yl)methoxy)phenyl)-5-(2-methoxypyridin-3-yl)-3-(pyridin-3-yl)pyrimidine-2,4-dione **31** (160 mg, 0.285 mmol) afforded 1-(3-chloro-5-((1-methyl-5-nitro-1H-imidazol-2-yl)methoxy)phenyl)-5-(2-oxo-1,2-dihydropyridin-3-yl)-3-(pyridin-3-yl)pyrimidine-2,4-dione **42** (57 mg, 0.10 mmol, 37% yield) as an off white solid.

Rf = 0.50 (10% MeOH/DCM). **^{1}H NMR (400 MHz, DMSO) δ** 11.92 (br s, 1H), 8.69 (s, 1H), 8.61 (dd, *J* = 4.8, 1.6 Hz, 1H), 8.57 (dd, *J* = 2.4, 0.8 Hz, 1H), 8.10 (s, 1H), 8.02 (dd, *J* = 7.2, 2.1 Hz, 1H), 7.85 (ddd, *J* = 8.1, 2.5, 1.6 Hz, 1H), 7.57 (ddd, *J* = 8.1, 4.8, 0.8 Hz, 1H), 7.41 – 7.34 (m, 3H), 7.32 – 7.27 (m (app. t), 1H), 6.30 (dd, *J* = 7.2, 6.4 Hz, 1H), 5.40 (s, 2H), 3.95 (s, 3H). **^{13}C NMR (101 MHz, DMSO) δ** 161.7, 160.9, 158.6, 149.6, 149.5, 149.2, 147.1, 143.3, 141.1, 140.1, 139.8, 136.8, 134.4, 133.8, 132.7, 131.6, 124.0, 121.3, 120.3, 115.1, 113.4, 108.0, 105.1, 62.7, 33.7. **HRMS**: (APCI+) $[M+H]^+$ calc. for $C_{25}H_{19}ClO_6N_7$, 548.1085, observed, 548.10797.

<u>Synthesis of 1-[3-Chloro-5-[(2-chlorophenyl)methoxy]phenyl]-5-(2-oxo-1H-pyridin-3-yl)-3-(3-pyridyl)pyrimidine-2,4-dione (**43**)</u>

Carried out according to *General procedure C*: Using 1-[3-chloro-5-[(2-chlorophenyl)methoxy]phenyl]-5-(2-methoxy-3-pyridyl)-3-(3-pyridyl)pyrimidine-2,4-dione (380 mg, 0.694 mmol) **32** afforded 1-[3-chloro-5-[(2-chlorophenyl)methoxy]phenyl]-5-(2-oxo-1*H*-pyridin-3-yl)-3-(3-pyridyl)pyrimidine-2,4-dione **43** (194 mg, 0.364 mmol, 52% yield).

^{1}H NMR (400 MHz, DMSO) δ 11.91 (s, 1H), 8.72 (s, 1H), 8.65 – 8.56 (m, 2H), 8.04 (dd, *J* = 7.1, 2.1 Hz, 1H), 7.85 (ddd, *J* = 8.1, 2.5, 1.5 Hz, 1H), 7.68 – 7.61 (m, 1H), 7.60 – 7.51 (m, 2H), 7.47 – 7.29 (m, 6H), 6.34 – 6.26 (m, 1H), 5.23 (s, 2H). **^{13}C NMR (101 MHz, DMSO) δ** 161.7, 160.9, 159.3, 149.6, 149.5, 149.1, 143.4, 141.2, 140.0, 136.8, 134.3, 133.9, 133.5, 132.9, 132.8, 130.6, 130.3, 129.5, 127.5, 124.0, 121.3, 119.8, 115.1, 112.9, 107.9, 105.1, 67.8. **HRMS**: (APCI+) $[M+H]^+$ calc. for $C_{27}H_{19}Cl_2N_4O_4$, 533.07779, observed, 533.07802.

<u>Synthesis of 1-(3-Chloro-5-((1-methyl-3-(trifluoromethyl)-1H-pyrazol-4-yl)methoxy)phenyl)-5-(2-oxo-1,2-dihydropyridin-3-yl)-3-(pyridin-3-yl)pyrimidine-2,4-dione (**44**)</u>

Carried out according to *General procedure C*: Using 1-[3-chloro-5-[[1-methyl-3-(trifluoromethyl)pyrazol-4-yl]methoxy]phenyl]-5-(2-methoxy-3-pyridyl)-3-(3-pyridyl)pyrimidine-2,4-dione **33** (134 mg, 0.229 mmol) afforded 1-(3-chloro-5-((1-methyl-3-(trifluoromethyl)-1H-pyrazol-4-yl)methoxy)phenyl)-5-(2-oxo-1,2-dihydropyridin-3-yl)-3-(pyridin-3-yl)pyrimidine-2,4-dione **44** (75 mg, 0.13 mmol, 57%) as a white solid.

^{1}H NMR (600 MHz, DMSO) δ 11.92 (s, 1H), 8.73 (s, 1H), 8.61 (dd, J = 4.8, 1.5 Hz, 1H), 8.58 – 8.56 (m, 1H), 8.12 (s, 1H), 8.03 (dd, J = 7.2, 2.1 Hz, 1H), 7.85 (ddd, J = 8.1, 2.4, 1.6 Hz, 1H), 7.59 – 7.55 (m, 1H), 7.38 (dd, J = 6.3, 2.1 Hz, 1H), 7.31 (t, J = 1.8 Hz, 1H), 7.25 – 7.23 (m, 2H), 6.31 – 6.27 (m, 1H), 5.10 (s, 2H), 3.92 (s, 3H). **^{13}C NMR (151 MHz, DMSO) δ** 161.7, 161.0, 159.0, 149.6, 149.5, 149.1, 143.4, 141.1, 139.9, 138.5, 138.3, 136.8, 134.4, 134.2, 133.9, 132.7, 124.0, 122.5, 121.2, 120.7, 119.6, 115.1, 114.5, 112.8, 107.9, 105.1, 60.0. **HRMS** (APCI) m/z calculated for $C_{26}H_{19}O_4N_6{}^{35}ClF_3$ $[M+H]^+$ 571.1103 found 571.1105. **^{19}F NMR** (565 MHz, DMSO) δ -59.47.

Synthesis of 1-(3-Chloro-5-((4-(trifluoromethyl)thiazol-5-yl)methoxy)phenyl)-5-(2-oxo-1,2-dihydropyridin-3-yl)-3-(pyridin-3-yl)pyrimidine-2,4-dione (**45**)

Carried out according to *General procedure C*: Using 1-[3-chloro-5-[[4-(trifluoromethyl)thiazol-5-yl]methoxy]phenyl]-5-(2-methoxy-3-pyridyl)-3-(3-pyridyl)pyrimidine-2,4-dione **34** (152 mg, 0.259 mmol) afforded 1-(3-chloro-5-((4-(trifluoromethyl)thiazol-5-yl)methoxy)phenyl)-5-(2-oxo-1,2-dihydropyridin-3-yl)-3-(pyridin-3-yl)pyrimidine-2,4-dione **45** (100 mg, 0.174 mmol, 67% yield) as a white solid.

^{1}H NMR (500 MHz, DMSO) δ 11.91 (s, 1H), 9.27 (s, 1H), 8.70 (s, 1H), 8.62 (dd, *J* = 4.8, 1.6 Hz, 1H), 8.57 (dd, *J* = 2.4, 0.8 Hz, 1H), 8.03 (dd, *J* = 7.1, 2.1 Hz, 1H), 7.84 (ddd, *J* = 8.1, 2.5, 1.6 Hz, 1H), 7.57 (ddd, *J* = 8.1, 4.8, 0.8 Hz, 1H), 7.40 – 7.33 (m, 3H), 7.31 (app t, *J* = 2.1 Hz, 1H), 6.30 (dd, *J* = 7.2, 6.4 Hz, 1H), 5.62 – 5.57 (m, 2H). **^{13}C NMR (151 MHz, DMSO) δ** 161.7, 160.9, 158.4, 156.4, 149.6, 149.5, 149.1, 143.3, 141.2, 134.0, 139.7, 139.5, 139.3, 139.0, 137.6, 137.6, 136.8, 134.3, 134.0, 132.7, 124.0, 123.8, 122.0, 121.3, 120.4, 120.2, 118.4, 115.1, 113.3, 108.0, 105.1, 61.7. **^{19}F NMR (565 MHz, DMSO) δ** -59.62. **HRMS** (APCI) m/z calculated for $C_{25}H_{16}O_4N_5{}^{35}ClF_3{}^{32}S$ $[M+H]^+$ 574.0558 found 574.0556.

Synthesis of 1-(3-(Benzyloxy)-5-chlorophenyl)-5-(6-methoxypyridin-3-yl)pyrimidine-2,4(1*H*,3*H*)-dione (**46**)

A Schlenk tube was charged with 5-(6-methoxypyridin-3-yl)pyrimidine-2,4(1*H*,3*H*)-dione **18** (950 mg, 4.33 mmol), followed by 1-(benzyloxy)-3-chloro-5-iodobenzene **21** (1.24 g, 3.60 mmol), K_3PO_4 (1.60 mg, 7.54 mmol), CuI (70 mg, 0.37 mmol) and *N*-(2-cyanophenyl)picolinamide (Ligand*) (96 mg, 0.43 mmol). The vessel was evacuated and back filled with argon. DMSO (25 mL) was added and the reaction mixture was degassed for 15 min by bubbling argon into the solution. The reaction was stirred overnight at 60 °C. Once cooled, the reaction solution was diluted with EtOAc and washed with water. The aqueous layer was then extracted with EtOAc (x3). The combined organic layers were then washed with $Cu(OAc)_2.H_2O$ (x2), 15% NH_4Cl (x2), brine and dried over $MgSO_4$. After filtration, the solution was concentrated *in vacuo* and then DCM was added, resulting in the formation of a precipitate. This was collected and used without further purification to afford 1-(3-(benzyloxy)-5-chlorophenyl)-5-(6-methoxypyridin-3-yl)pyrimidine-2,4(1*H*,3*H*)-dione **46** (285 mg, 0.654 mmol, 18%) as an off-white solid.

Rf = 0.55 (80% EtOAc/Hexane). **^{1}H NMR (600 MHz, DMSO) δ** 11.74 (br s, 1H), 8.39 (s, 1H), 8.00 – 7.97 (m, 1H), 7.95 – 7.89 (m, 1H), 7.49 – 7.43 (m, 2H), 7.43 – 7.39 (m, 2H), 7.37 – 7.33 (m, 1H), 7.26 (dq, *J* = 15.9, 1.8 Hz, 2H), 7.22 (q, *J* = 1.9 Hz, 1H), 6.84 (d, *J* = 8.6 Hz, 1H), 5.17 (s, 2H), 3.86 (s, 3H). **^{13}C NMR (151 MHz, DMSO) δ** 162.8, 162.6, 159.3, 149.6, 145.9, 141.9, 140.5, 139.1, 136.2, 133.6, 128.5, 128.1, 127.9, 122.0, 119.7, 114.6, 113.3, 110.6, 109.7, 70.0, 53.2. **HRMS**: (APCI+) $[M+H]^+$ calc. for $C_{23}H_{19}ClO_4N_3$, 436.1064, observed, 436.10566.

Scheme S4: Synthesis towards target compound 51

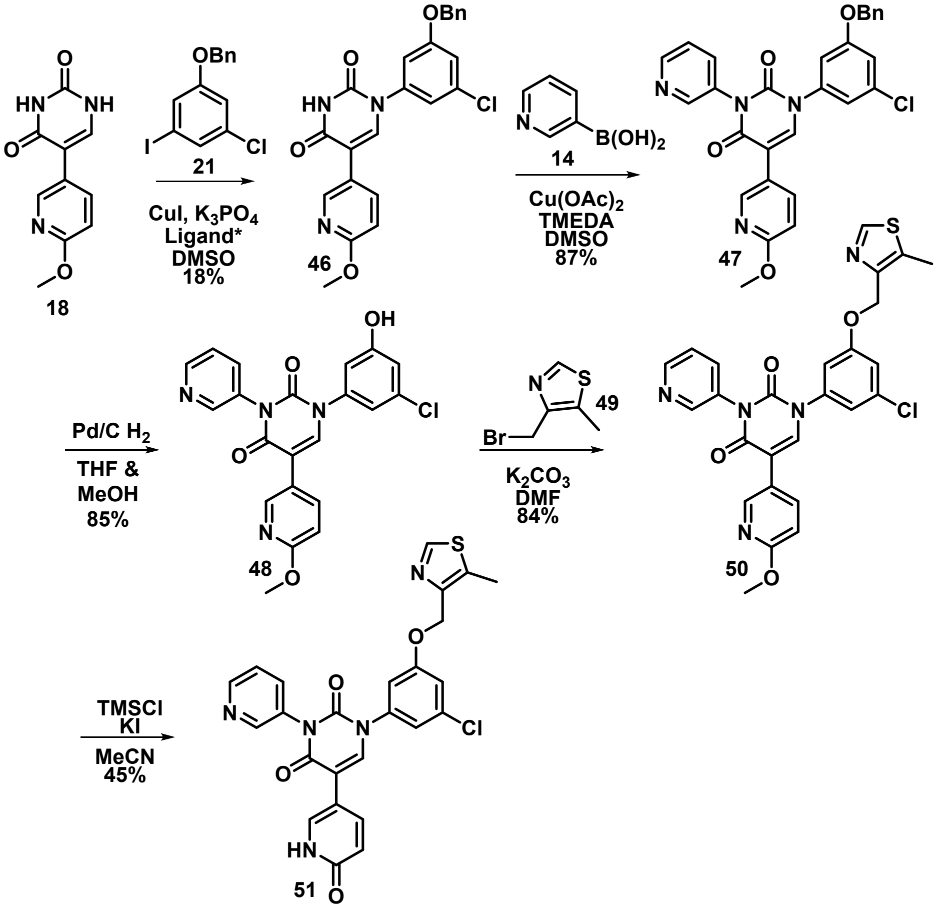

<u>Synthesis of 1-(3-(Benzyloxy)-5-chlorophenyl)-5-(6-methoxypyridin-3-yl)-3-(pyridin-3-yl)pyrimidine-2,4(1*H*,3*H*)-dione (**47**)</u>

To a round-bottomed flask was added 1-(3-(benzyloxy)-5-chlorophenyl)-5-(6-methoxypyridin-3-yl)pyrimidine-2,4(1*H*,3*H*)-dione **46** (265 mg, 0.609 mmol), followed by 3-pyridinyl boronic acid **14** (749 mg, 6.09 mmol) and DMSO (6 mL). Copper(II) acetate (221 mg, 1.22 mmol) was then added to the reaction solution followed by TMEDA (0.91 mL, 6.1 mmol). The reaction mixture was stirred at 60 °C open to the atmosphere and monitored using LCMS. Once completed, EtOAc was added and the solution was washed with H_2O (x2), brine and dried over $MgSO_4$. Filtration was then followed by *in vacuo* removal of solvent and purification by column chromatography (EtOAc/Hex) to afford 1-(3-(benzyloxy)-5-chlorophenyl)-5-(6-methoxypyridin-3-yl)-3-(pyridin-3-yl)pyrimidine-2,4(1*H*,3*H*)-dione **47** (270 mg, 0.526 mmol, 87%) as an off-white solid.

Rf = 0.55 (80% EtOAc/Hexane). **^{1}H NMR (600 MHz, DMSO) δ** 8.64 – 8.61 (m, 1H), 8.60 – 8.58 (m, 1H), 8.45 – 8.40 (m, 1H), 8.20 (s, 1H), 7.96 (dd, *J* = 8.7, 2.4 Hz, 1H), 7.89 – 7.83 (m, 1H), 7.57 (dd, *J* = 8.1, 4.8 Hz, 1H), 7.48 – 7.35 (m, 5H), 7.34 (d, *J* = 1.6 Hz, 1H), 7.31 (s, 1H), 7.25 (s, 1H), 6.86 (d, *J* = 8.7 Hz, 1H), 5.18 (s, 2H), 3.87 (s, 3H). **^{13}C NMR (151 MHz, DMSO) δ** 162.9, 161.8, 159.4, 149.8, 149.6, 149.2, 146.1, 141.5, 140.8, 139.3, 136.8, 136.2, 133.7, 132.6, 128.5 (2C), 128.1, 127.9 (2C), 124.0, 122.1, 119.7, 114.8, 113.3, 110.4, 109.78, 70.1, 53.2. **HRMS**: (APCI+) $[M+H]^+$ calc. for $C_{28}H_{22}ClO_4N_4$, 513.1330, observed, 513.13249.

Synthesis of 1-(3-Chloro-5-hydroxyphenyl)-5-(6-methoiixypyridin-3-yl)-3-(pyridin-3-yl)pyrimidine-2,4(1*H*,3*H*)-dione (**48**)

Into two neck round bottom flask containing argon was placed 11-(3-(benzyloxy)-5-chlorophenyl)-5-(6-methoxypyridin-3-yl)-3-(pyridin-3-yl)pyrimidine-2,4(1*H*,3*H*)-dione **47** (254 mg, 0.495 mmol) followed by methanol (3 mL) and THF (3 mL). With heating, the starting material solubilised and remained in solution after cooling. To this was added Pd/C (11 mg, 0.099 mmol) and a balloon filled with hydrogen was fitted to the flask. The atmosphere in the flask was purged with hydrogen gas and the reaction was left to proceed at 40 °C. After three hours, analysis by LCMS revealed that all of the starting material had been converted to the desired product and so the reaction mixture was filtered through celite and concentrated *in vacuo* to afford 1-(3-chloro-5-hydroxyphenyl)-5-(6-methoxypyridin-3-yl)-3-(pyridin-3-yl)pyrimidine-2,4(1*H*,3*H*)-dione **48** (177 mg, 0.419 mmol, 85%) as a white solid which was used without further purification or characterisation other than LCMS.

Synthesis of 1-(3-Chloro-5-((5-methylthiazol-4-yl)methoxy)phenyl)-5-(6-methoxypyridin-3-yl)-3-(pyridin-3-yl)pyrimidine-2,4(1H,3H)-dione (**50**)

Carried out according to *General procedure A* using: 1-(3-chloro-5-hydroxyphenyl)-5-(6-methoxypyridin-3-yl)-3-(pyridin-3-yl)pyrimidine-2,4(1*H*,3*H*)-dione **48** (177 mg, 0.419 mmol), 4-(chloromethyl)-5-methyl-1,3-thiazole hydrochloride **49** (154 mg, 0.839 mmol), K_2CO_3 (232 mg, 1.68 mmol), DMF (10 mL). Afforded 1-(3-chloro-5-((5-methylthiazol-4-yl)methoxy)phenyl)-5-(6-methoxypyridin-3-yl)-3-(pyridin-3-yl)pyrimidine-2,4(1H,3H)-dione **50** (187 mg, 0.350 mmol, 71%) as a pale yellow solid.

Rf = 0.25 (5% MeOH/DCM). **^{1}H NMR (600 MHz, DMSO) δ** 8.90 (s, 1H), 8.63 – 8.61 (m, 1H), 8.60 – 8.58 (m, 1H), 8.44 – 8.41 (m, 1H), 8.21 (s, 1H), 7.98 – 7.94 (m, 1H), 7.88 – 7.85 (m, 1H), 7.57 (dd, *J* = 8.1, 4.8 Hz, 1H), 7.34 (d, *J* = 1.7 Hz, 1H), 7.30 – 7.29 (m, 2H), 6.86 (d, *J* = 8.7 Hz, 1H), 5.22 (s, 2H), 3.86 (s, 3H). **^{13}C NMR (151 MHz, DMSO) δ** 162.9, 161.8, 159.4, 151.1, 149.8, 149.6, 149.2, 147.1, 146.1, 141.5, 140.7, 139.3, 136.8, 133.6, 133.3, 132.6, 124.0, 122.1, 119.8, 114.8, 113.3, 110.4, 109.8, 63.9, 53.3, 10.7. **HRMS**: (APCI+) $[M+H]^+$ calc. for $C_{26}H_{21}ClO_4N_5S$, 534.1003, observed, 534.0996. Note that in the ^{1}H NMR the thiazole methyl protons are missing and are suspected to be hidden behind the DMSO signal. This methyl's ^{13}C NMR signal is present and the HRMS is consistent with the correct structure.

Synthesis of 1-(3-Chloro-5-((5-methylthiazol-4-yl)methoxy)phenyl)-5-(6-oxo-1,6-dihydropyridin-3-yl)-3-(pyridin-3-yl)pyrimidine-2,4(1*H*,3*H*)-dione (**51**)

Carried out according to *General procedure C* using: 1-(3-chloro-5-((5-methylthiazol-4-yl)methoxy)phenyl)-5-(6-methoxypyridin-3-yl)-3-(pyridin-3-yl)pyrimidine-2,4(1*H*,3*H*)-dione **50** (170 mg, 0.319 mmol), NaI (120 mg, 0.797 mmol), TMSCl (87 mg, 0.80 mmol), MeCN (8 mL). Afforded 1-(3-chloro-5-((5-methylthiazol-4-yl)methoxy)phenyl)-5-(6-oxo-1,6-dihydropyridin-3-yl)-3-(pyridin-3-yl)pyrimidine-2,4(1*H*,3*H*)-dione **51** as an off-white solid (75 mg, 0.15 mmol, 45%).

Rf = 0.25 (5% MeOH/DCM). **^{1}H NMR (600 MHz, DMSO) δ** 11.73 (br s, 1H), 8.90 (s, 1H), 8.61 (d, *J* = 4.8, 1.4 Hz, 1H), 8.57 (d, *J* = 2.4 Hz, 1H), 8.14 (s, 1H), 7.84 (d, *J* = 8.2 Hz, 1H), 7.78 (d, *J* = 2.7 Hz, 1H), 7.74 (dd, *J* = 9.6, 2.7 Hz, 1H), 7.56 (dd, *J* = 8.2, 4.8 Hz, 1H), 7.34 – 7.26 (m, 3H), 6.36 (d, *J* = 9.6 Hz, 1H), 5.22 (s, 2H), 3.34 (s, 3H). **^{13}C NMR (151 MHz, DMSO) δ** 161.7, 161.6, 159.4, 151.1, 149.7, 149.6, 149.2, 147.1, 141.1, 140.7, 140.3, 136.8, 134.3, 133.6, 133.3, 132.6, 124.0, 119.8, 119.3, 114.7, 113.4, 110.3, 109.5, 63.9, 10.7. **HRMS**: (APCI+) $[M+H]^+$ calc. for $C_{25}H_{19}ClO_4N_5S$, 520.0846, observed, 520.08400.

Scheme S5: Synthesis towards precursors 55 and 57

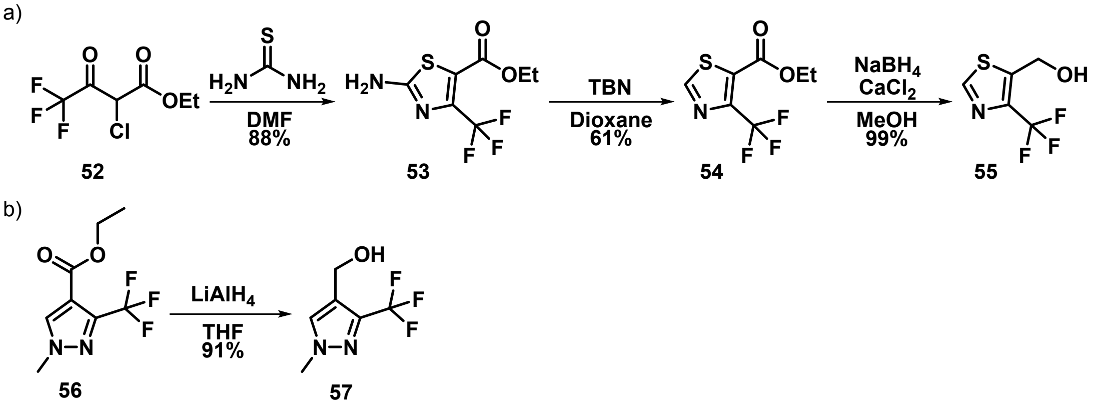

Synthesis of Ethyl 2-amino-4-(trifluoromethyl)thiazole-5-carboxylate (**53**)

Ethyl 2-chloro-4,4,4-trifluoro-3-oxo-butanoate **52** (2.00 g, 9.15 mmol, 1.44 mL) and thiourea (2.09 g, 27.5 mmol) were dissolved in DMF (12 mL) and heated to 120 °C for 5 hours. The reaction was allowed to cool to room temperature and diluted with EtOAc (100 mL). The organic phase was washed with deionized water (3 x 100 mL) followed by a wash with brine and dried over $MgSO_4$. The solvent was reduced *in vacuo* and the crude purified over silica gel using EtOAc/hexanes as eluent to produce ethyl 2-amino-4-(trifluoromethyl)thiazole-5-carboxylate **53** (1.93 g, 88% yield) as a yellow solid.

^{1}H NMR (500 MHz, DMSO) δ 8.24 (s, 2H), 4.21 (q, *J* = 7.1 Hz, 2H), 1.24 (t, *J* = 7.1 Hz, 3H).

Synthesis of Ethyl 4-(trifluoromethyl)thiazole-5-carboxylate (**54**)

A solution of ethyl 2-amino-4-(trifluoromethyl)thiazole-5-carboxylate **53** (1.93 g, 8.02 mmol) in dioxane (23 mL) was heated to 90 °C. To this was added *tert*-butyl nitrite (2.4 mL, 18 mmol) dropwise, taking precautions due to the rapid evolution of nitrogen gas. After gas evolution had subsided, the reaction was allowed to stir for an additional 40 minutes and then cooled. The solvent was removed *in vacuo* and the crude material purified by column chromatography (EtOAc/Hex) to afford ethyl 4-(trifluoromethyl)thiazole-5-carboxylate **54** as a yellow liquid (1.11 g, 61% yield).

^{1}H NMR (600 MHz, $CDCl_3$) δ 8.88 (s, 1H), 4.41 (q, *J* = 7.1 Hz, 2H), 1.39 (t, *J* = 7.2 Hz, 3H).

Synthesis of [4-(Trifluoromethyl)thiazol-5-yl]methanol (**55**)

To a solution of ethyl 4-(trifluoromethyl)thiazole-5-carboxylate **54** (1.11 g, 4.92 mmol) in methanol (22 mL) and water (8 mL) was added $CaCl_2$ (0.710 g, 6.40 mmol). The solution was cooled to 0 °C after the complete dissolution of the solids, $NaBH_4$ (0.483 g, 12.8 mmol) was added in portions. The reaction was allowed to stir at room temperature overnight, after which the reaction was naturalized by the addition of aqueous saturated sodium bicarbonate solution. The product was extracted with DCM (2 x 100 mL), the organic phase washed with brine and dried over $MgSO_4$. After filtration, the solvent was removed, and the crude [4-(trifluoromethyl)thiazol-5-yl]methanol **55** (0.900 g, 99% yield) was used in the next reaction without purification.

^{1}H NMR (500 MHz, $CDCl_3$) δ 7.91 (s, 1H), 4.23 – 4.21 (m, 2H), 3.23 – 3.06 (m, 1H).

Synthesis of (1-Methyl-3-(trifluoromethyl)-1H-pyrazol-4-yl)methanol (**57**)

To as solution of ethyl 1-methyl-3-(trifluoromethyl)pyrazole-4-carboxylate **56** (0.500 mg, 2.25 mmol) in THF (11 mL) at 0 °C, was added $LiAlH_4$ (188 mg, 4.95 mmol) and the reaction was allowed to stir at that temperature for 3 h. The reaction was quenched with saturated sodium bicarbonate solution and washed with EtOAc (2 x 50 mL). The organic phase was washed with brine, dried over $MgSO_4$ and the solvent reduced *in vacuo* to afford (1-methyl-3-(trifluoromethyl)-1H-pyrazol-4-yl)methanol **57** (367 mg, 91% yield) which was used without further purification in the next step.

^{1}H NMR (500 MHz, $CDCl_3$) δ 7.38 (s, 1H), 4.56 (s, 2H), 3.84 (s, 3H), 3.15 (s, 1H).

Molecular Modelling:

Docking studies were carried out using Glide-SP (Schrödinger modelling suite, versions 2020-1 to 2022-4). Advanced settings changes included using enhanced sampling (4x) and increasing the settings under Selection of initial poses (50000, 1000, 4000 resp. and "use expanded sampling"). The maximum number of minimisation steps was increased to 1000. When docking was used for the purposes of preparing structures for FEP analysis, the same settings were employed but with the additional setting of using core constraints such that all ligands were restricted to placement of a common core and only the S4 pocket moiety conformationally sampled. Relative free energy binding values were initially obtained using the FEP protocol within Desmond (DE Shaw Research group) and then later using FEP+ from Schrödinger. For the purposes of docking and FEP analysis, initially the SARS-CoV-2 Mpro receptor PDB ID 7N44 was utilised, however this was changed to 7M8N later in the project due to improved FEP+ predictability. In both cases, only chain A was used for FEP even though the active form is the homodimer (retrospective analysis of both the monomers and homodimers did not afford superior results when using the homodimer and so the monomers were used going forward due to significantly reduced calculation times). In all cases, receptors were prepared using the Protein Preparation workflow, including adding missing side chains (and optimisation thereof) as necessary.

Enzymatic and Whole Cell Assay Procedures:

Enzymatic (IC_{50}) Assay Protocol

Reaction Buffer:
50 mM Tris-HCl pH 7.3, 1 mM EDTA, 0.005% Triton X-100, 1% DMSO, +/- 1 mM DTT.

Enzymes:
SARS-CoV-2 Mpro (3CLpro): Reaction Biology Corp. product, MSC-11-519. Recombinant protease (GenBank accession: QHD43415, aa3264-3569) expressed in *E. coli* with no tag, MW = 33.8 kDa. 25 μM in reaction.

Substrate:
Covidyte ED450: AAT Bioquest Cat# 13538; [NH2-C(EDANS)VNSTQSGLRK(DABCYL)M-COOH] FRET peptide substrate. MW=2,034 Da. 5 μM in reaction.

Control compound:
GC376

Measurement: EnVision (PE). Ex/Em 340/492 nm

Reaction Procedure: Prepare indicated enzyme and substrate in freshly prepared reaction buffer. Deliver enzyme solution into the reaction well. Then deliver compounds in DMSO into the reaction mixture by using Acoustic Technology (Echo 550, LabCyte Inc. Sunnyvale, CA) in nanoliter range. After 20 min pre-incubation, deliver substrate solution into the reaction well to initiate the reaction. The enzyme activities were monitored every 5 min as a time-course measurement of the increase in fluorescence signal from fluorescently-labelled peptide substrate for 120 min. at room temperature.

Data Analysis: Take slope (signal/time) of linear portion of measurement. Slope is calculated using Excel, and curve fits are performed using Prism software. The IC_{50} curve fits were performed using GraphPad Prism 4 software with 4 parameters fit using the following formula:

*Y = Bottom + (Top-Bottom)/(1+10^((LogIC50-X)*HillSlope))*

Prism setting: 4 parameters sigmoidal dose-response (variable slope), constraint; Bottom = 0, Top less than 120.

Curve fits were performed when the % enzyme activities at the highest concentration of compounds were less than 65%.

Whole Cell (EC_{50}) Assay Protocol

Cells and viruses

African green monkey kidney epithelial cells constitutively expressing human TMPRSS2 (VeroE6/TMPRSS2; JCRB Cell Bank, CVCL_YQ49) were maintained in Dulbecco's modified Eagle medium (DMEM) supplemented with 7.5% fetal bovine serum (Corning). SARS-CoV-2 WA-1 expressing NanoLuc luciferase (SARS-CoV-2-NLuc) was amplified on VeroE6/TMPRSS2 cells (MOI = 0.001). Infectious titers were determined by $TCID_{50}$ titration.

SARS-CoV-2-NLuc reporter virus dose response assays

For dose response assays, compound was added in 3-fold dilutions (0.009–20 μM) to 96 well plates seeded with VeroE6/TMPRSS2cells (1.1×10^4/well), followed by infection with SARS-CoV-2-NLuc reporter virus (MOI = 0.2 $TCID_{50}$ units/cell) and automated plate reading 28 hours after infection. Assays were performed in triplicate for each compound (3 independent repeats, each consisting of 3 technical repeats). Four-parameter variable slope regression (Graphpad Prism 9.4.1) was used to determine EC_{50} and CC_{50} concentrations.

Metabolic Stability Assay

Liver Microsome Stability Assay Protocol

Human liver microsomes (HLMs, 20 mg/mL), CD-1 mouse liver microsomes (MLMs, 20 mg/mL), Sprague Dawley rat liver microsomes (RLMs, 20 mg/mL) were purchased from Xenotech. NADPH was purchased from Sigma-Aldrich and prepared in 10 mM stock solutions of distilled H_2O (Invitrogen^{TM} UltraPure^{TM}). Verapamil and diphenhydramine were both purchased from Sigma-Aldrich and served as positive controls for LM stability assays. Test compounds and positive controls were initially dissolved in DMSO to make 10 mM stock solutions. Sample solutions were then further diluted in 100% MeCN to 500 µM. Next, the reactions were prepared by mixing human or mouse liver microsomes (55 µL) with potassium phosphate buffer (100 mM, 928 µL) in 1.5 mL Eppendorf tubes. The test compounds (6.6 µL of 500 µM solution) were subsequently added to the suspensions, and the reaction mixtures were incubated at 37 ^{o}C for 5 min. Afterward, the liver microsome reactions were initiated with 110 µL of 10 mM NADPH and further incubated at 37 ^{o}C for the designated time course of the study. This procedure provided experiments with a final volume of 1100 µL (0.6% organic solvent content) and a final test compound concentration of 3 µM. Aliquots (100 µL) were removed from each reaction mixture in duplicate at 0, 5, 10, 15, and 30 min time intervals and quenched with 100 µL of cold internal standard solution (ISTD, 2 µM 7-ethoxy-d_5-coumarin in MeOH or MeCN). Quenched aliquots were then centrifuged at 12,500 g for 5-10 min, and the resulting supernatants were withdrawn and placed in LC-MS vials to be analyzed by LC-MS/MS (Agilent G6460C QQQ MS coupled with an Infinity II 1260 HPLC). Each test compound was run in tandem with positive and negative control experiments for quality assurance. Positive control reactions were conducted at a final volume of 550 µL for a single run of each time point. Lastly, the negative control experiment was conducted with test compounds and liver microsomes in the absence of NADPH (150 µL) and analyzed at the 30 min time point.

Data Analysis. Each data point was analyzed in triplicate using in-between blank washes to avoid carry over and to equilibrate the column for the subsequent runs. Averages of these triplicates for individual compounds at each time point were then normalized to the data at 0 min, representing 100% test compound remaining or 0% metabolism. Half-lives ($t_{1/2}$) were calculated by plotting ln of % test compound remaining versus time and performing linear regression to determine slope. Slope = −k and $t_{1/2}$ = 0.693/k for first-order kinetics (see Figures S1-S15).

Liver Microsome Stability Assay Setup Example:

Test Compound: 928.4 µL Potassium Phosphate Buffer (100 mM) + 55 µL HLM or MLM + 110 µL NADPH (10 mM) + 6.6 µL TC (500 µM).

Positive Control: 464.2 µL Potassium Phosphate Buffer (100 mM) + 27.5 µL HLM or MLM + 55 µL NADPH (10 mM) + 3.3 µL Verapamil or Diphenhydramine (500 µM).

Negative Control: 141.6 µL Potassium Phosphate Buffer (100 mM) + 7.5 µL HLM or MLM + 0.9 µL TC (500 µM).

Quenching Mixture: 100 µL MeCN or MeOH with ISTD (2 µM d5-7-ethoxy coumarin).

Final Volume After Quenching: 200 μL (100 μL from reaction mixture + 100 μL quencher solution; ISTD final concentration of 1.0 μM).

Instrumentation and LC-MS/MS Method Development

LC-MS/MS analyses were conducted on an Agilent 1260 Infinity II HPLC equipped with an Agilent G6460 triple quadrupole mass spectrometer (Agilent Technologies, USA). Reverse-phase HPLC separation for each compound was achieved with an Agilent InfinityLab Poroshell 120 EC-C18 (2.1 x 50 mm, 2.7 μm) or EC-C8 (2.1 x 50 mm, 2.7 μm) column maintained at 40 ^{o}C. The mobile phase used during analyses consisted of either MeOH-H_2O (0.1% FA) or MeCN-H_2O (0.1% FA) at a flow rate of 0.5 mL/min. Each method was developed in the presence of the internal standard (ISTD), 7-ethoxy-d_5-coumarin. Precursor and product ion detection was performed with Agilent Jet Stream electrospray positive ionization (ESI+) in multiple reaction monitoring (MRM) mode. All MRM transitions, fragmentor voltages, and collision energies for individual compounds are provided in **Table S2**. Other standard MS conditions were as follows: dwell time of 100 ms; gas flow of 10 L/min; nebulizer pressure of 45 psi; delta EMV of 200 V. Lastly, all acquired data was processed using Agilent 6460 Quantitative Analysis software.

Table S1: Scan parameters and corresponding transitions in MRM mode

Compound	Precursor Ion MS1	Product Ion MS2	Fragmentor Voltage (V)	Collision Energy (V)	Cell Accelerator (V)	Polarity
**4**	565.1	440.0<br>125.0	170	33<br>41	4	Positive
**43**	533.1	413.0<br>125.0	80	21<br>45	4	Positive
**36**	505.1	385.1<br>121.1	158	25<br>50	4	Positive
**41**	539.1	419.2<br>131.1<br>121.1	170	21<br>37<br>49	4	Positive
**Diphenhydramine**	256.2	167.1<br>152.0	78	8<br>44	4	Positive
**Verapamil**	455.1	303.2<br>165.1	126	24<br>28	4	Positive
**Procaine**	237.2	120.0<br>100.2	98	25<br>13	4	Positive
**Enalapril**	377.2	234.2<br>117.1	116	17<br>41	4	Positive
**7-Ethoxy-d_5-coumarin (ISTD)**	196.1	164.0	116	17	4	Positive

Liver Microsome Stability Data of Selected Compounds

Table S2: Liver microsome stability as % remaining (30 min) and half-life ($t_{1/2}$)

	4	36	41	43	45
HLM	4.9%	91.7%	70.7%	48%	19.5%
($t_{½}$)	(5.6)	(>30)	(>30)	(27.8)	(13)
MLM	0.7%	73.3%	22.8%	4.0%	14.4%
($t_{½}$)	(2.4)	(>30)	(14.7)	(3.0)	(11)
RLM	1.4%	86.4%	90.2%	26.6%	30.9%
($t_{½}$)	(4.8)	(>30)	(>30)	(16.1)	(18)

Figure S1: Stability of 4 in human liver microsomes (HLM) at 37 °C

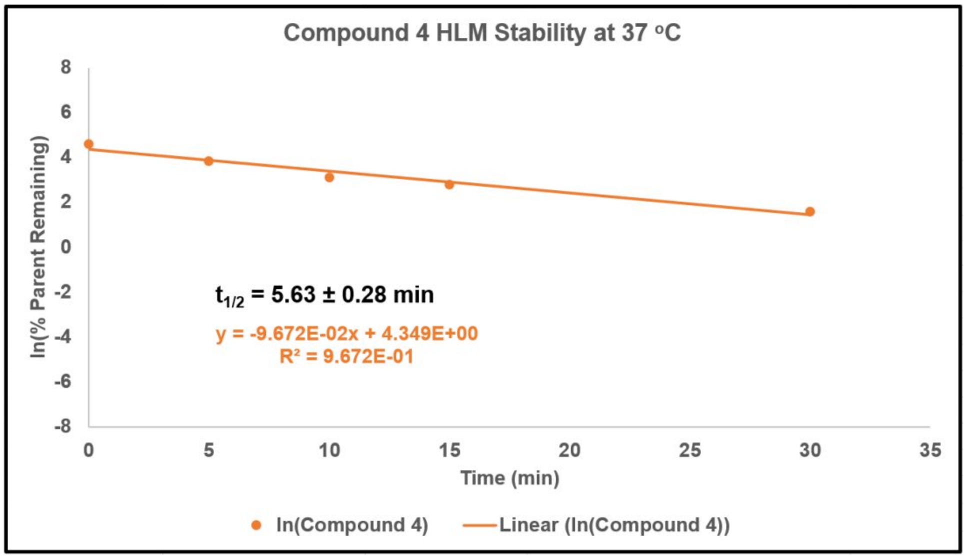

Table S3: Stability of 4 in human liver microsomes (HLM) at 37 °C

Time	Response ratio A	Response ratio B	Average RR	Rel %	ln
0	1.33	1.50	1.42	100	4.605170186
5	0.62	0.69	0.65	46.08	3.830305497
10	0.31	0.33	0.32	22.67	3.120842226
15	0.23	0.23	0.23	16.28	2.78998087
30	0.07	0.07	0.07	4.92	1.593624686

Figure S2: Stability of 4 in mouse liver microsomes (MLM) at 37 °C

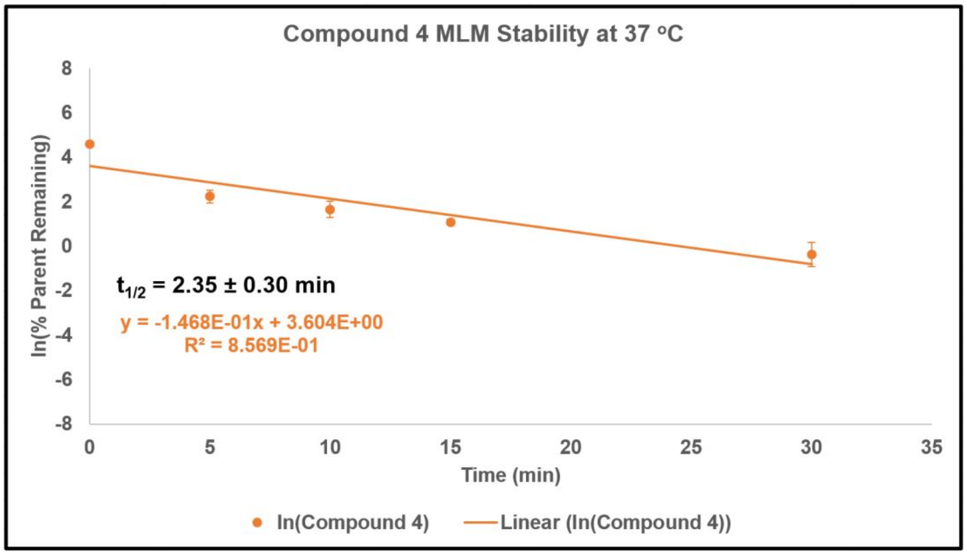

Table S4: Stability of 4 in mouse liver microsomes (MLM) at 37 °C

Time	Response ratio		Average RR	Rel %	ln
	A	B			
0	0.66	0.47	0.56	100	4.605170186
5	0.052	0.055	0.053	9.41	2.241271701
10	0.027	0.032	0.030	5.25	1.657485792
15	0.018	0.015	0.017	2.96	1.084302809
30	0.00	0.00	0.00	0.69	-0.376406038

Figure S3: Stability of 4 in rat liver microsomes (RLM) at 37 °C

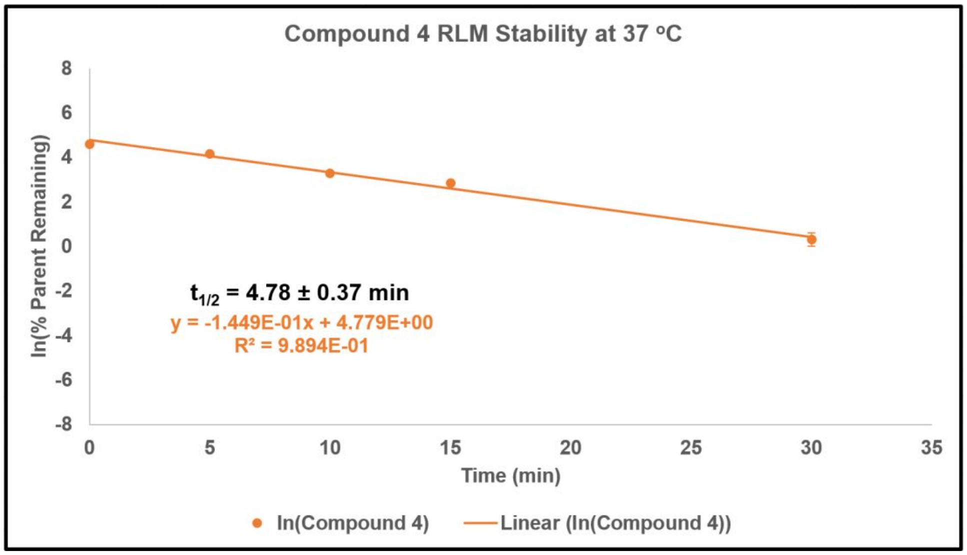

Table S5: Stability of 4 in rat liver microsomes (RLM) at 37 °C

Time	Response ratio A	Response ratio B	Average RR	Rel %	ln
0	1.14	1.22	1.18	100	4.605170186
5	0.73	0.78	0.75	63.57	4.152083912
10	0.32	0.30	0.31	26.57	3.279824084
15	0.19	0.22	0.21	17.35	2.853865796
30	0.012	0.020	0.016	1.36	0.306564666

Figure S4: Stability of 36 in human liver microsomes (HLM) at 37 °C

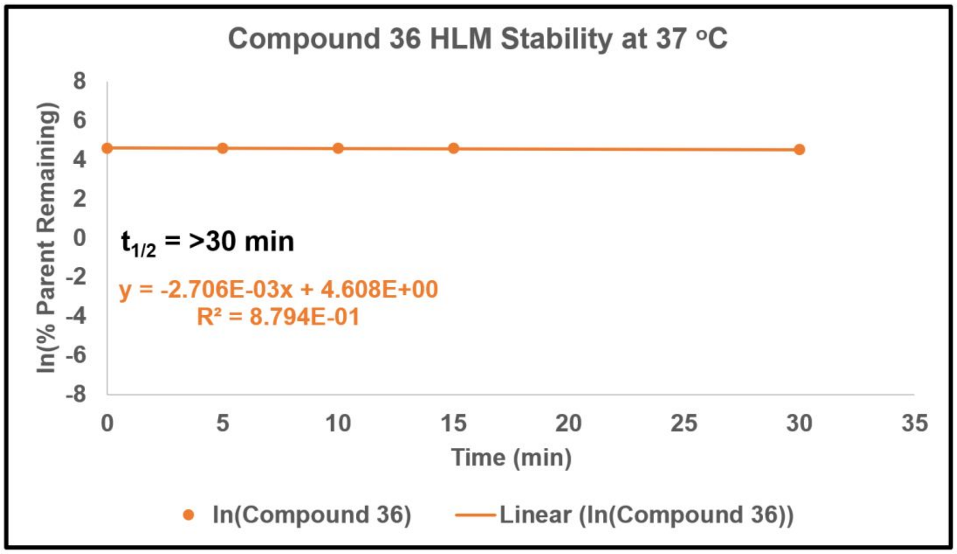

Table S6: Stability of 36 in human liver microsomes (HLM) at 37 °C

Time	Response ratio A	Response ratio B	Average RR	Rel %	ln
0	2.29	2.29	2.29	100	4.605170186
5	2.24	2.25	2.25	98.15	4.152083912
10	2.24	2.24	2.24	97.71	3.279824084
15	2.23	2.27	2.25	98.22	2.853865796
30	2.10	2.098	2.10	91.71	0.306564666

Figure S5: Stability of 36 in mouse liver microsomes (MLM) at 37 °C

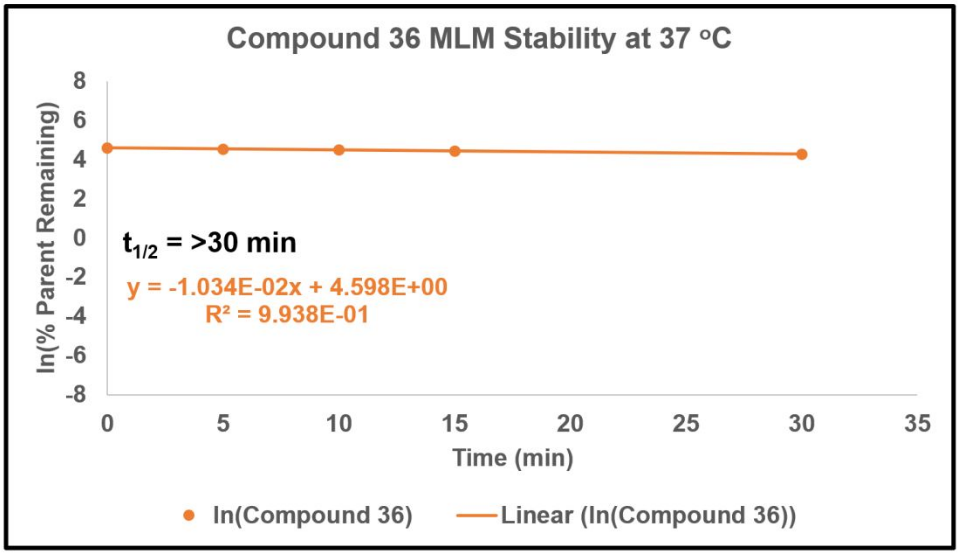

Table S7: Stability of 36 in mouse liver microsomes (MLM) at 37 °C

Time	Response ratio A	Response ratio B	Average RR	Rel %	ln
0	2.66	2.75	2.71	100	4.605170186
5	2.57	2.51	2.54	93.89	4.542108076
10	2.45	2.42	2.44	90.09	4.500843565
15	2.26	2.28	2.27	83.80	4.428460667

30	1.99	1.98	1.98	73.26	4.293966711

Figure S6: Stability of 36 in rat liver microsomes (RLM) at 37 °C

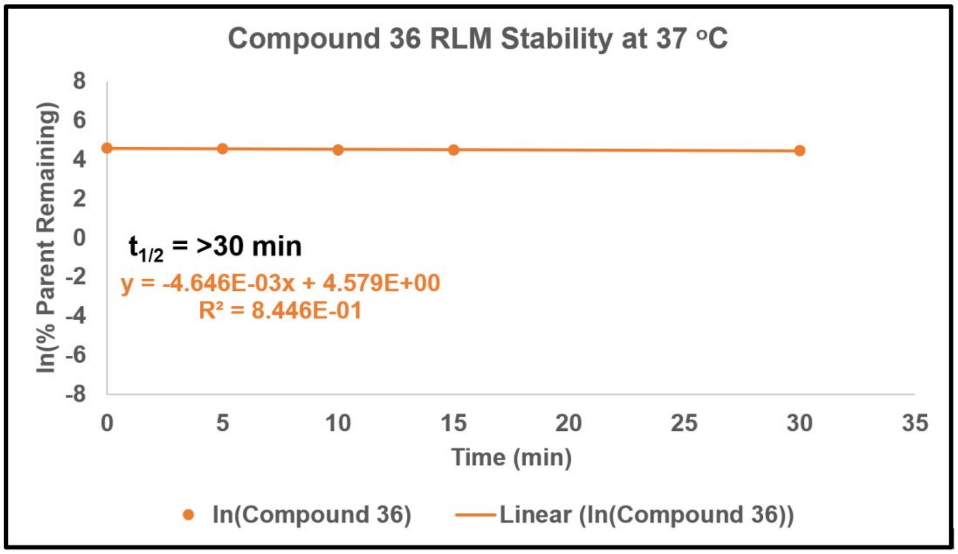

Table S8: Stability of 36 in rat liver microsomes (RLM) at 37 °C

Time	Response ratio		Average RR	Rel %	ln
	A	B			
0	3.48	3.50	3.49	100	4.605170186
5	3.25	3.41	3.33	95.37	4.557810727
10	3.14	3.21	3.17	90.89	4.509636557
15	3.12	3.08	3.10	88.82	4.486657055
30	3.01	3.02	3.02	86.41	4.459078909

Figure S7: Stability of 41 in human liver microsomes (HLM) at 37 °C

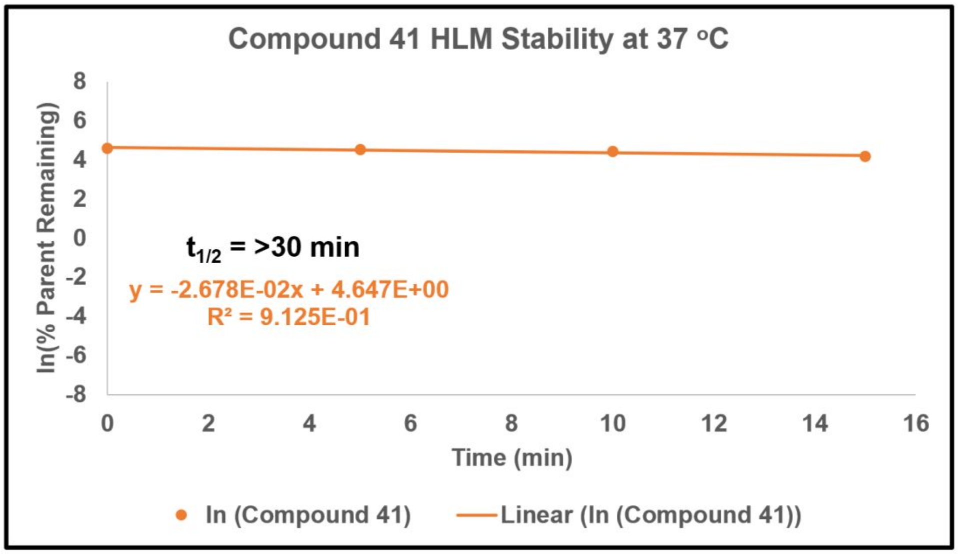

Table S9: Stability of 41 in human liver microsomes (HLM) at 37 °C

Time	Response ratio		Average RR	Rel %	ln
	A	B			
0	1.61	1.51	1.56	100	4.605170186
5	1.52	1.42	1.47	94.46	4.557810727
10	1.29	1.34	1.32	84.43	4.509636557
15	1.04	1.04	1.04	66.44	4.486657055
30	1.11	1.10	1.10	70.70	4.459078909

Figure S8: Stability of 41 in mouse liver microsomes (MLM) at 37 °C

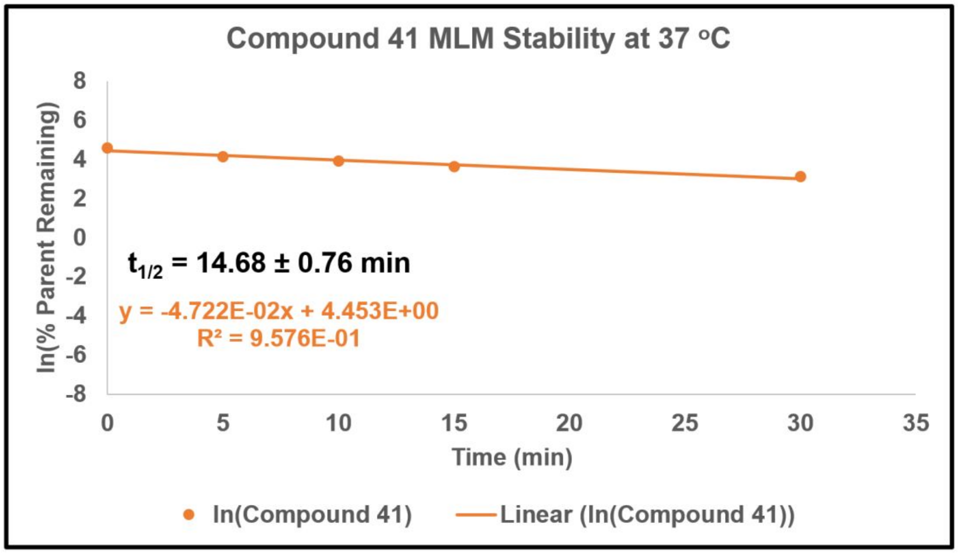

Table S10: Stability of 41 in mouse liver microsomes (MLM) at 37 °C

Time	Response ratio A	Response ratio B	Average RR	Rel %	ln
0	0.91	0.93	0.92	100	4.605170186
5	0.59	0.58	0.58	63.80	4.155678011
10	0.47	0.45	0.46	50.25	3.917010456
15	0.36	0.33	0.35	37.70	3.629593684
30	0.22	0.20	0.21	22.79	3.126183985

Figure S9: Stability of 41 in rat liver microsomes (RLM) at 37 °C

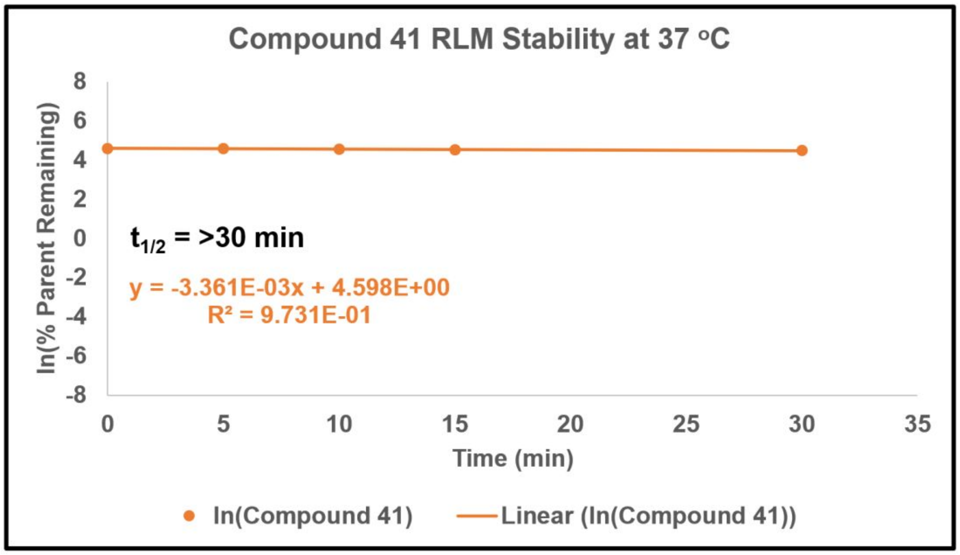

Table S11: Stability of 41 in rat liver microsomes (RLM) at 37 °C

Time	Response ratio A	Response ratio B	Average RR	Rel %	ln
0	0.96	0.98	0.97	100	4.605170186
5	0.95	0.94	0.95	97.59	4.580777885
10	0.92	0.92	0.92	95.21	4.556064168
15	0.91	0.92	0.91	93.93	4.542579513
30	0.88	0.87	0.88	90.17	4.501703462

Figure S10: Stability of 43 in human liver microsomes (HLM) at 37 °C

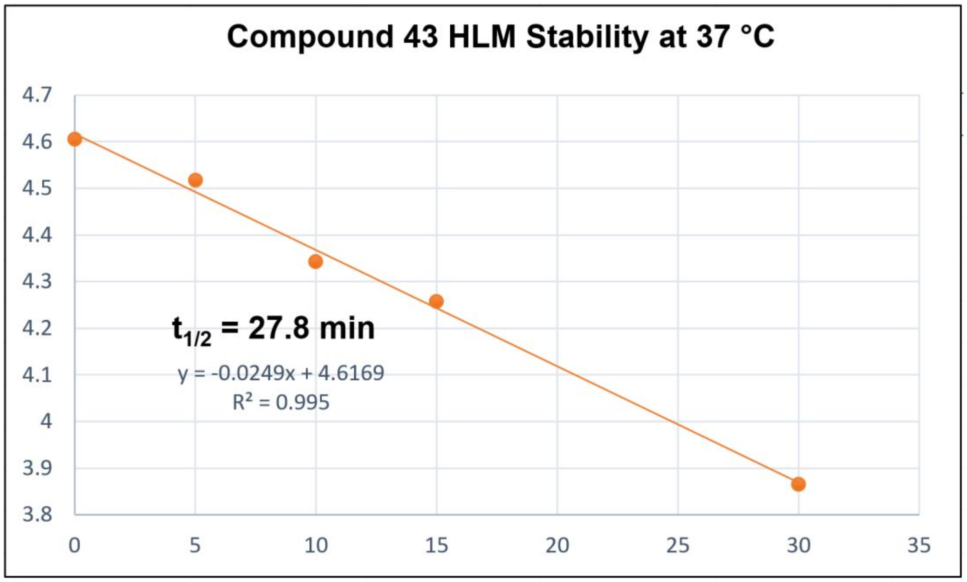

Table S12: Stability of 43 in human liver microsomes (HLM) at 37 °C

Time	Prodrug AUC A	Prodrug AUC B	Average AUC	Rel %	ln
0	140517	146292	143405	100	4.60517019
5	133667	129081	131374	91.61	4.51754783
10	106181	114320	110251	76.88	4.34225593
15	104768	97899	101334	70.66	4.25791958
30	67731	69149	68440	47.72	3.86545346

Figure S11: Stability of 43 in mouse liver microsomes (MLM) at 37 °C

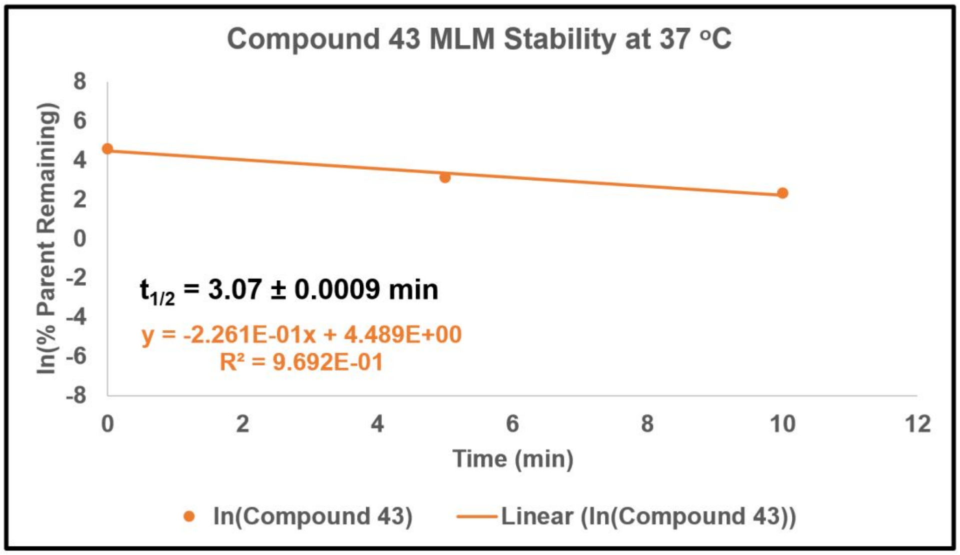

Table S13: Stability of 43 in mouse liver microsomes (MLM) at 37 °C

Time	Response ratio		Average RR	Rel %	ln
	A	B			
0	1.70	1.77	1.73	100	4.605170186
5	0.39	0.40	0.40	22.77	3.125500446
10	0.18	0.18	0.18	10.43	2.344298175
15	0.13	0.13	0.13	7.53	2.018840335
30	-	0.07	0.07	3.96	1.376680202

Figure S12: Stability of 43 in rat liver microsomes (RLM) at 37 °C

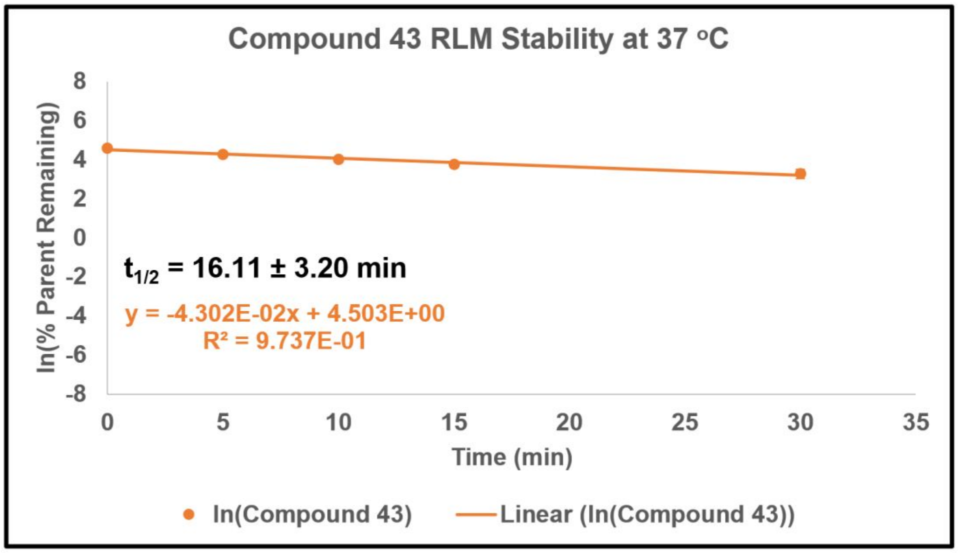

Table S14: Stability of 43 in rat liver microsomes (RLM) at 37 °C

Time	Response ratio		Average RR	Rel %	ln
	A	B			
0	2.35	1.58	1.96	100	4.605170186
5	1.60	1.19	1.40	71.12	4.26443424
10	1.21	0.97	1.09	55.30	4.0128198
15	0.85	-	0.85	43.45	3.771592181
30	0.54	0.51	0.52	26.55	3.279105044

Figure S13: Stability of 45 in human liver microsomes (HLM) at 37 °C

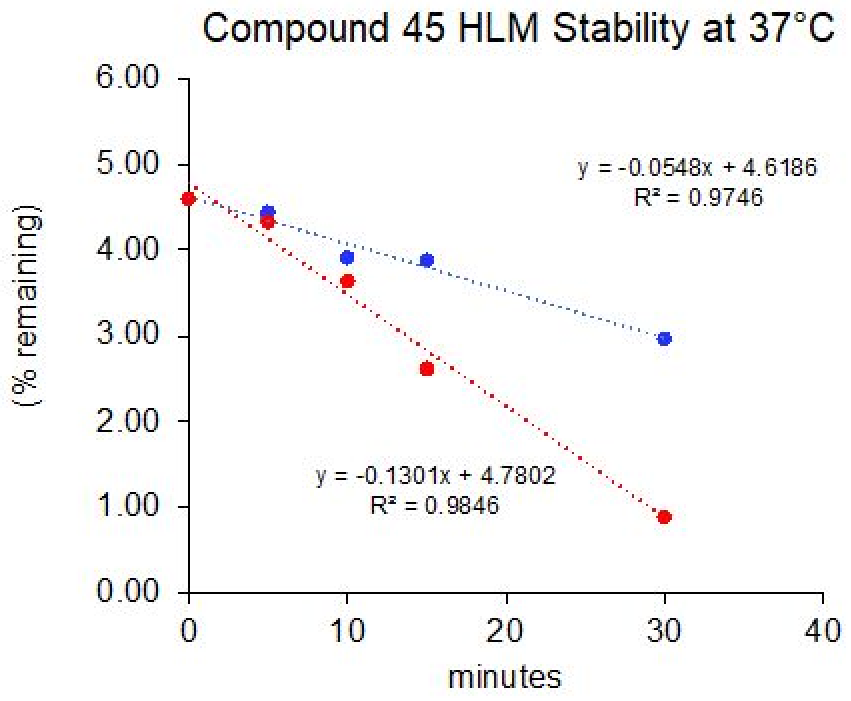

Table S15: Stability of 45 in human liver microsomes (HLM) at 37 °C

Time	Response ratio A	Response ratio B	Average RR	Rel %	ln
0	2.07	1.88	1.98	100	4.61
5	1.70	1.64	1.67	84.56	4.44
10	-	0.99	0.99	49.92	3.91
15	0.96	0.96	0.96	48.45	3.88
30	0.34	0.43	0.38	19.47	2.97

Figure S14: Stability of 45 in mouse liver microsomes (MLM) at 37 °C

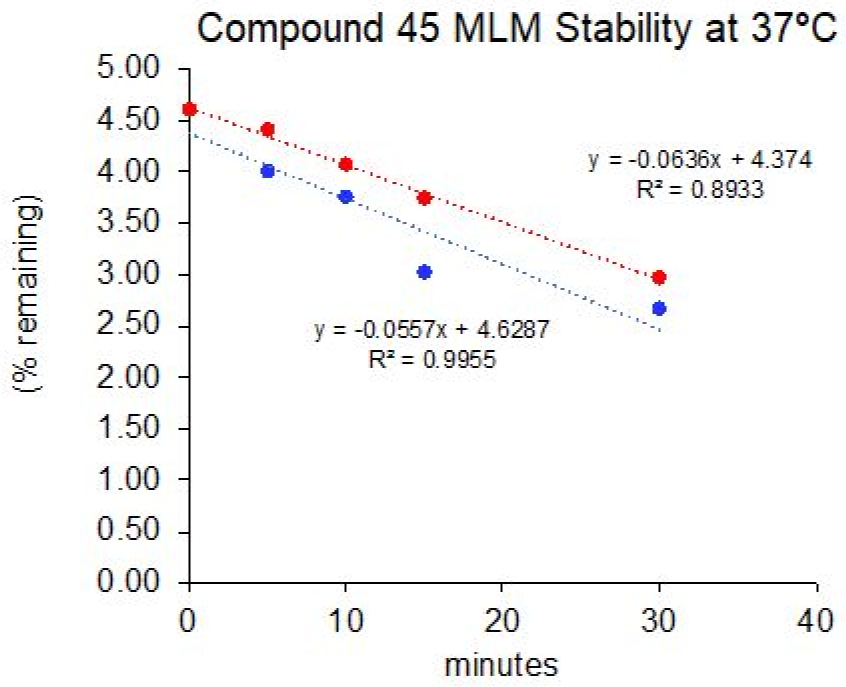

Table S16: Stability of 45 in mouse liver microsomes (MLM) at 37 °C

Time	Response ratio		Average RR	Rel %	ln
	A	B			
0	1.38	1.35	1.36	100	4.61
5	0.75	0.74	0.74	54.6	4.00
10	0.56	0.61	0.59	42.96	3.76
15	0.27	0.29	0.28	20.55	3.02
30	0.18	0.21	0.20	14.41	2.67

Figure S15: Stability of 45 in rat liver microsomes (RLM) at 37 °C

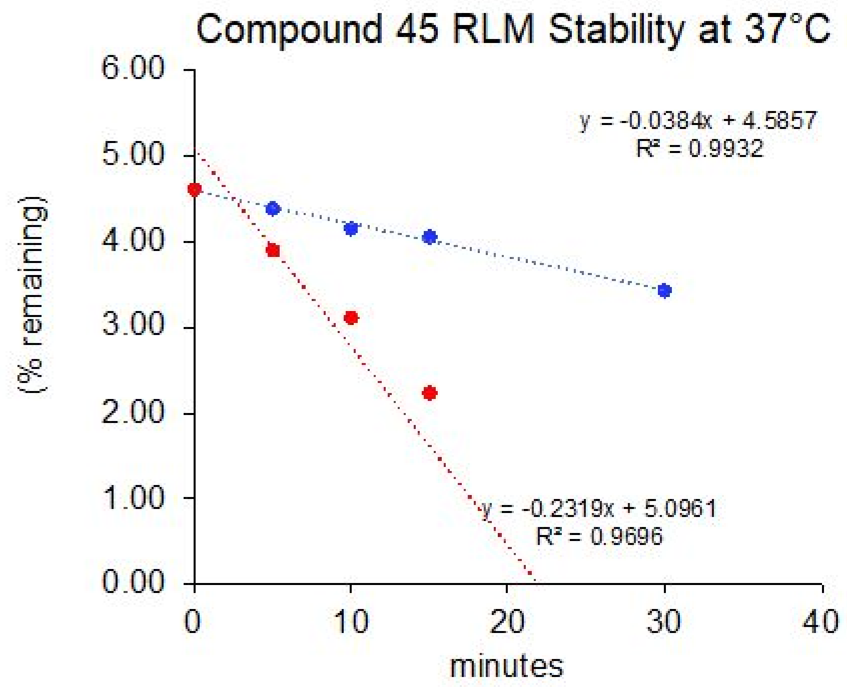

Table S17: Stability of 43 in rat liver microsomes (RLM) at 37 °C

Time	Response ratio		Average RR	Rel %	ln
	A	B			
0	2.51	2.84	2.68	100	4.61
5	1.90	2.40	2.15	80.16	4.38
10	1.57	1.83	1.70	63.43	4.15
15	1.57	1.53	1.55	57.78	4.06
30	0.86	0.79	0.83	30.88	3.43

Metabolite identification

***Procedure for Liver Microsome Metabolite Identification Assay*:** Test compounds and positive controls were dissolved in 100% DMSO to make 10 mM stock solutions. The 10 mM stock solutions of test and control

compounds were further diluted in potassium phosphate buffer (100 mM, pH 7.4) to 500 µM to ensure that the organic solvent content was < 0.2%. The liver microsome (HLM or MLM) assay was prepared in a 1.5 mL Eppendorf tube with a final volume of 1100 µL for duplicate runs. Each reaction contained phosphate buffer (374 µL), liver microsomes (550 µL), and test compound (66 µL of 500 µM), resulting in a final concentration of 30 µM for the test compound. The reaction was initiated with 110 µL of 10 mM NADPH. Aliquots (100 µL) were removed in duplicate at 0, 5, 10, 15, and 30 min and quenched in 200 µL of 100 % cold acetonitrile which contained internal standard (ISTD: *d5*-7-ethoxy coumarin 2 µM). The aliquots were centrifuged at 13,000 g for 5 min, and the supernatant was transferred into autosampler vials for LC-HRMS analysis.

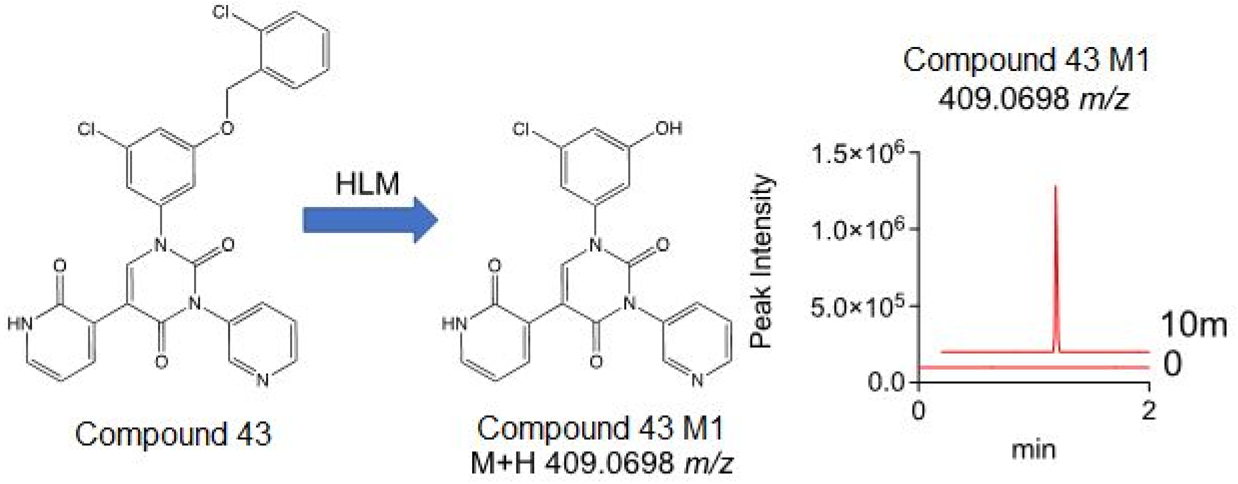

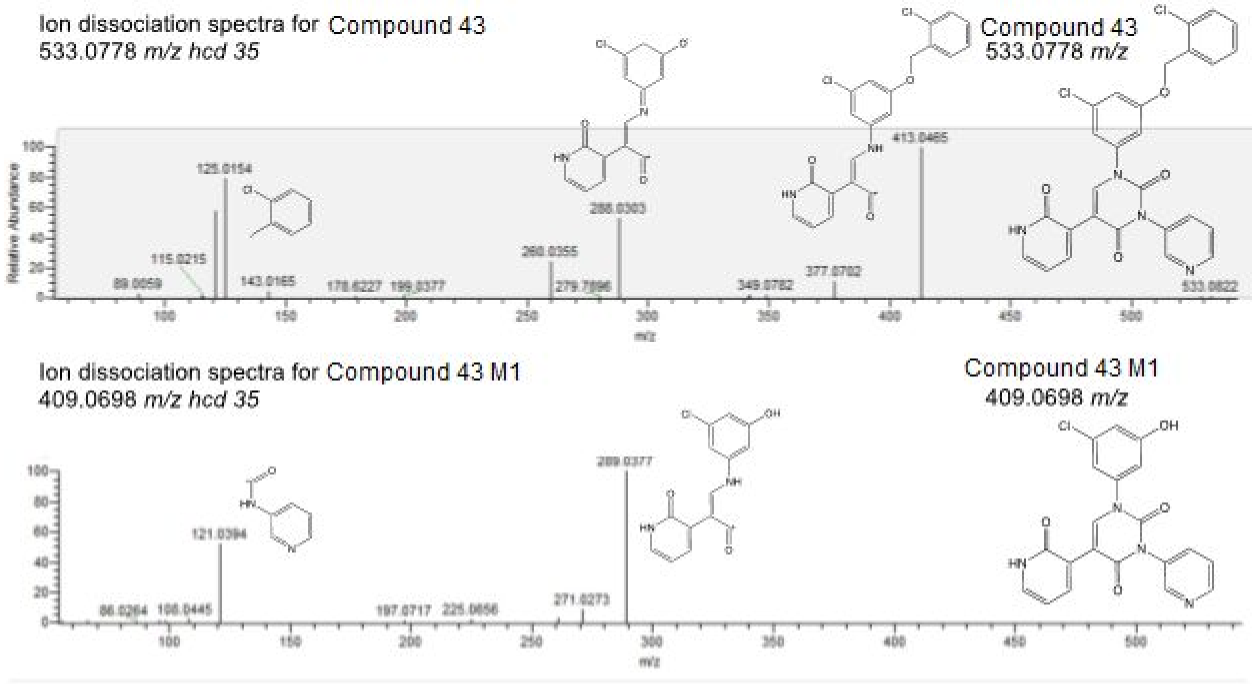

Figure S16: Metabolite identification study of Compound 43 in liver microsomes using liquid chromatography coupled to high resolution mass spectrometry (LC-HRMS). The identification of metabolite Compound 43 M1 (409.0698 m/z) within 10 min is indicative of ether cleavage in liver microsomes.

MS analysis was performed using full scan from a mass range of 100-700 m/z at 60k resolution with data dependent MS/MS and MSn programs enabled for the top 5 ions detected for each scan. Compounds are detected based on their MS1 precursor ion accurate mass *m/z* and identified with matching theoretical and observed accurate mass MS1 ions with interpretation of accurate mass MS/MS spectra from expected metabolites.

Pharmacokinetic Studies

Table S18: Pharmacokinetic data of Compound 36 in Male Sprague Dawley rats following a single intravenous and oral administration (Dose: 0.25 mg/kg, IV and 0.5 mg/kg, PO)

Matrix	Route	Dose (mg/kg)	T_{max} (h)	$^{a}C_0/C_{max}$ (ng/mL)	AUC_{last} (h*ng/mL)	AUC_{inf} (h*ng/mL)	$T_{1/2}$ (h)	CL (mL/min /kg)	V_{ss} (L/kg)	%F
Plasma	IV	0.25	-	1326.56	1611.56	1676.66	1.71	2.49	0.33	-
	PO	0.50	1.67	284.94	1307.99	1687.40	-	-	-	**40.58**

[a] – Back extrapolated concentration in IV group.

Figure S17: Plasma concentrations-time profiles (mean ± SD) of Compound 36 in Male Sprague Dawley rats following a single intravenous and oral administration (Dose: 0.25 mg/kg, IV and 0.5 mg/kg, PO)

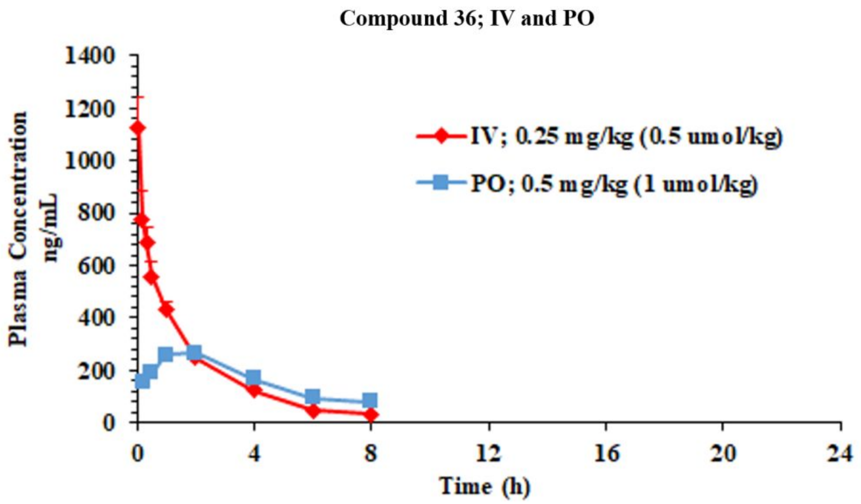

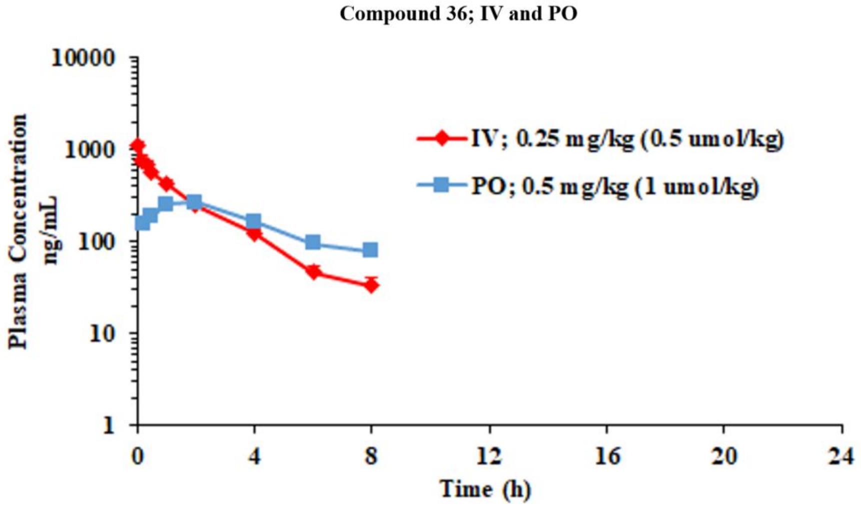

Table S19. Summary of Pharmacokinetic Parameters (nmol/mL) for Compound 36 in Rat Plasma after a Single Oral Dose of 36 at 10 mg/kg or 100 mg/kg

Dose (mg/kg)	T_{max} (h)	C_{max} (nmol/mL)	T_{last} (h)	C_{last} (nmol/mL)	AUC_{inf} (h*nmol/mL)	$t_{1/2}$ (h)
PO-10	1.67 ± 0.577	2.61 ± 0.476	24.0 ± 0.00	0.0126 ± 0.00627	23.2 ± 2.18	2.68 ± 0.310
PO-100	2.33 ± 1.53	4.13 ± 0.161	24.0 ± 0.00	0.114 ± 0.105	49.9 ± 4.53	3.73 ± 1.08

Results are shown as averages and standard deviations (n = 3).

Figure S18. Average Concentrations (nmol/mL) of Compound 36 in Rat Plasma after a Single Oral Dose of 36 at 10 mg/kg or 100 mg/kg

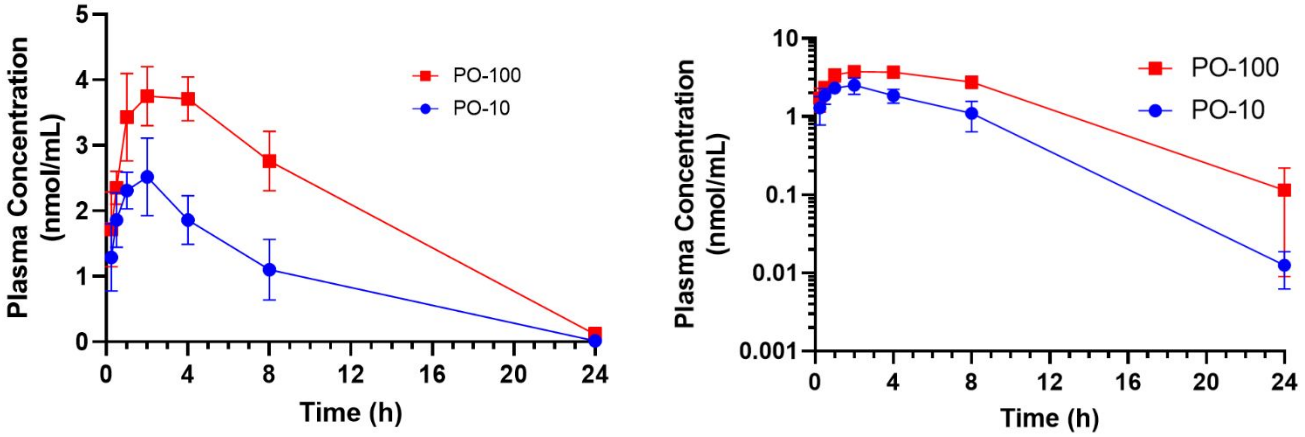

Left panel: Linear Scale, Right panel: Log Scale. Results are shown as averages and standard deviations (n = 3).

Figure S19: Figure of all final compounds tested

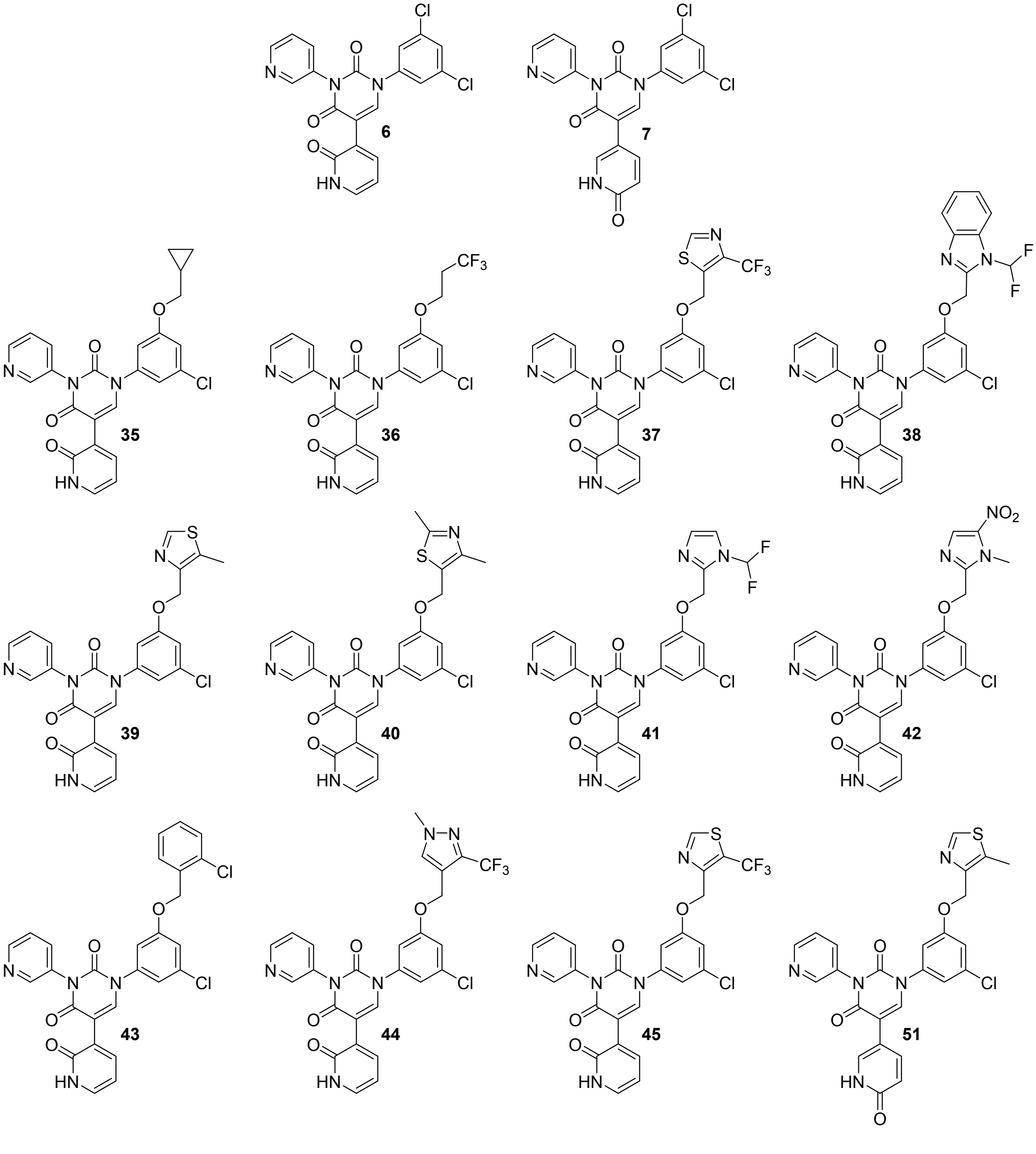

Supplement: Supplementary file 1 — ml3c00335_si_001.pdf [file ml3c00335_si_001.pdf]
